# Social Sampling and Expressed Attitudes: Authenticity Preference and Social Extremeness Aversion Lead to Social Norm Effects and Polarization

**DOI:** 10.1037/rev0000342

**Published:** 2022-01

**Authors:** Gordon D. A. Brown, Stephan Lewandowsky, Zhihong Huang

**Affiliations:** 1 Department of Psychology, University of Warwick; 2 School of Psychological Science, University of Bristol; 3 Department of Psychology, University of Western Australia

**Keywords:** polarization, social comparison, agent-based model, decision by sampling, social contagion

## Abstract

A cognitive model of social influence (Social Sampling Theory [SST]) is developed and applied to several social network phenomena including polarization and contagion effects. Social norms and individuals’ private attitudes are represented as distributions rather than the single points used in most models. SST is explored using agent-based modeling to link individual-level and network-level effects. People are assumed to observe the behavior of their social network neighbors and thereby infer the social distribution of particular attitudes and behaviors. It is assumed that (a) people dislike behaving in ways that are extreme within their neighborhood social norm (*social extremeness aversion* assumption), and hence tend to conform and (b) people prefer to behave consistently with their own underlying attitudes (*authenticity preference* assumption) hence minimizing dissonance. Expressed attitudes and behavior reflect a utility-maximizing compromise between these opposing principles. SST is applied to a number of social phenomena including (a) homophily and the development of segregated neighborhoods, (b) polarization, (c) effects of norm homogeneity on social conformity, (d) pluralistic ignorance and false consensus effects, (e) backfire effects, (f) interactions between world view and social norm effects, and (g) the opposing effects on subjective well-being of authentic behavior and high levels of social comparison. More generally, it is argued that explanations of social comparison require the variance, not just the central tendency, of both attitudes and beliefs about social norms to be accommodated.

Why does group discussion lead to polarization, why do people prefer to pay selective attention to opinions similar to their own, and why does exposure to neutral or even contradictory evidence sometimes strengthen pre-existing opinions? What cognitive mechanisms underpin social contagion effects, causing attitudes and behaviors to spread through social networks over time? Here, we examine how cognitive models of individuals’ context-based judgments can be applied to understanding of social norms, confirmation bias, polarization, and other social phenomena that occur at the level of social networks.

Social context has long been known to influence people’s behavior and the attitudes that people express (e.g., Festinger, 1954; Hyman, 1942). People compare themselves with others (Goethals & Darley, 1977) and often adjust their behavior in the direction of a social norm (see Buunk & Gibbons, 2007, for a review). The term “social norm” is used in a number of different ways (Bicchieri, 2006; Morris et al., 2015); here, we focus on descriptive social norms (Cialdini et al., 1991), which simply refer to what people are observed to do and say (i.e., the attitudes and beliefs people publicly express and the behaviors they engage in).^1^

More recently, social influence has been one of the key principles underpinning the concept of “nudging” as a method for guiding and influencing people’s behavior without restricting their freedom of choice (Thaler & Sunstein, 2008). The related idea that social contagion occurs, leading to the spread of behaviors such as smoking cessation, exercise levels, or excessive drinking through social networks (e.g., Aral & Nicolaides, 2017; Rosenquist et al., 2010), has attracted both attention and controversy. Other research has examined polarization both in the laboratory (see Sunstein, 2009, for a review) and on social media such as Twitter (Barberá et al., 2015; Garimella & Weber, 2017), with a particular interest in the relation between polarization, social media, and disinformation (Tucker et al., 2018). Additional lines of investigation search for the nature and causes of the increased political polarization that has occurred particularly in developed Western countries over recent decades (McCarty et al., 2006), while also identifying situations where polarization does not occur or may reduce (Baldassarri & Bearman, 2007; Boxell et al., 2017; Festinger, 1954; Fiorina & Abrams, 2008). In related work, recent laboratory studies have examined how judgments propagate along social chains (Moussaïd et al., 2017) and models of the social dynamics of risk transmission have been developed (Moussaïd et al., 2015).

The individual cognitive processes underlying these well-established social phenomena are however only poorly understood. Many models of social comparison processes have remained at the level of verbal expression and have not made contact with quantitative cognitive models of judgment and decision-making, reflecting a lack of cross talk between cognitive models of context-based judgment and social approaches (cf. Treat & Viken, 2010; we note some exceptions below).

Here, we present a computational account that we hope can start to bridge individual and social levels of description. We develop a quantitative model of social judgment and social influence at the level of the individual, based on independently motivated models of judgment and choice, and then use agent-based simulations to explore the model’s behavior at a network level. Our primary aim is to shed light on a wider number of known phenomena than previous models have been able to account for, and to do so in a way that respects well-established cognitive principles of individual judgment and decision-making. However the model also makes predictions that we believe to be novel, and we summarize these at the end of this paper as well as exploring the effects of varying model parameters as we describe the model’s behavior.

Our model (SST, for Social Sampling Theory) assumes that individuals have private attitudes and attitude-related beliefs that may not be consciously accessible to the individuals themselves and which may differ from the attitudes and beliefs that the individuals publicly express. SST assumes that individuals lose utility^2^ if their publicly expressed attitudes differ from the ones they privately hold. Why, then, would individuals ever express attitudes that differ from those that they privately hold? SST’s answer is that individuals infer norms in their social neighborhoods by observing the attitudes and attitude-related beliefs expressed by their network neighbors. These social norms are represented as distributions, and individuals also lose utility to the extent that their publicly expressed attitudes are extreme within the social distribution.

These two competing motivations—*authenticity preference* on the one hand and *social extremeness aversion* on the other—determine people’s public expressions of attitudes and attitude-related beliefs. In a nutshell, people want to be “true to themselves,” but they also do not want to be seen as “extreme” within their social networks. Thus, individuals’ choices about what attitudes to express in a given social environment reflect the tension that arises when there is a discrepancy between their private attitudes and the prevailing social norms.

The plan of the rest of this paper is as follows. First, we provide an intuitive description of the model. We then motivate the model’s assumptions, and locate it with respect to previous accounts. The majority of this paper is devoted to showing how the model accounts for specific phenomena.

## Intuitive Illustration of the Model

Much of the explanatory ability of SST derives from its assumption that both private attitudes and social norms are represented as distributions rather than single points; this assumption allows us to represent both the precision of private attitudes and the degree of consensus underlying social norms. Narrower (more sharply peaked) distributions represent more precise attitudes or greater social consensus. We argue below that this emphasis on distributions rather than single points is essential both to account for phenomena such as backfire and norm homogeneity effects and to render the model consistent with independent evidence for rank-based judgment.

**Figure 1 fig1:**
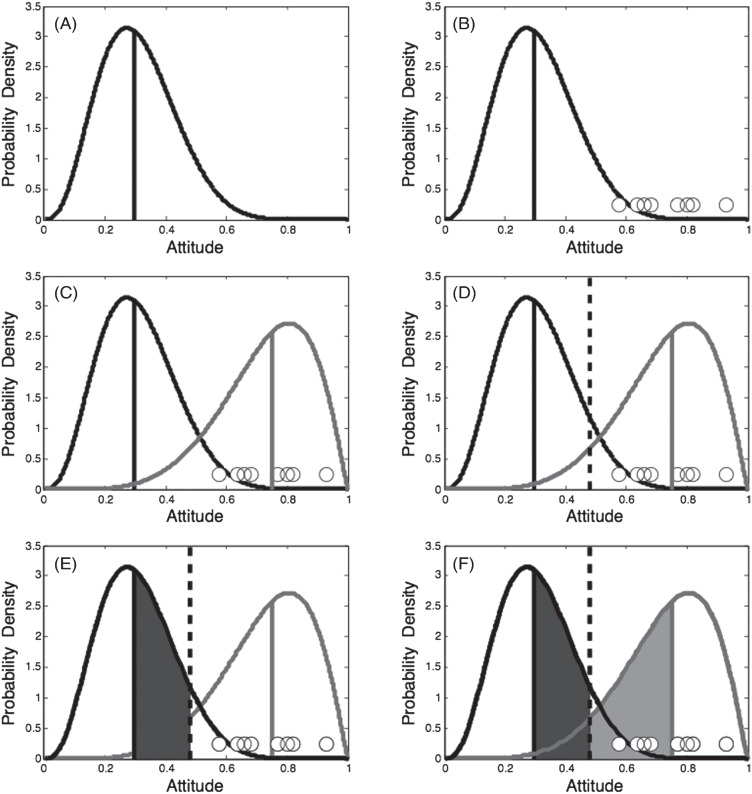
Intuitive Illustration of the Effect of Social Norms on Attitude Expressed by an Individual Agent (see Text for Details) *Note*. Solid lines represent the agent’s private attitude (vertical line shows median); circles represent attitudes expressed by social network neighbors; shaded lines represent the social norm inferred by the agent (vertical line shows median); vertical-dashed line shows the agent’s expressed attitude. The dark-shaded area represents the extent to which the expressed attitude departs from median authentic attitude; light-shaded area reflects extent of social extremeness.

The process of observing neighbors’ behaviors and inferring a social norm is illustrated in panels A through C of Figure 1. We illustrate with a hypothetical person, Alison, and (in this example, political) attitudes represented from zero (*left-wing/liberal*) to 1 (*right-wing/conservative*). Figure 1A illustrates Alison’s private attitude; her attitude is moderately liberal (median equals .3) and is held with a moderate degree of conviction (the distribution that represents her attitude is neither very narrow nor very wide). In almost all the simulations below, we assume that these private attitudes are fixed for each individual. We can think of the height of the curve at any point on the ideological continuum as expressing the extent to which an individual would endorse or accept that ideological position. The .3 (median) represents the attitude that Alison would express if she were uninfluenced by the attitudes expressed by other people. Alison’s private attitude is not visible either to other people or to herself.^3^ Alison also observes the political viewpoints expressed by her social network neighbors. (We assume she observes each neighbor just once.) The views expressed by Alison’s eight nearest neighbors are illustrated by the eight small circles lying just above the horizontal axis of Figure 1B. All of these views happen to fall to the right of center, and are clustered around a median of about .7. These observed attitudes inform Alison’s belief about the ideological social norm that prevails in her neighborhood.[Fig fig1]

Next, Alison infers the social norm by fitting (or at least behaving as if she is fitting) a distribution to the attitudes she has observed her neighbors expressing. The inferred social norm in this example is illustrated by the solid-gray line in Figure 1C. Thus Alison’s representation of the social norm—just like her representation of her own private attitude—is a distribution, not a single point. Her representation of the social norm is unbiased in the sense that it accurately reflects the attitudes expressed by her social network neighbors, but it will be a biased estimate of the wider population norm if her neighbors’ attitudes are not representative.

The political attitude that Alison will express is hypothesized to depend both on her own private attitude and on her perception of the social norm (neither of which is assumed to be consciously available). In intuitive terms, Alison will tend to express an attitude that is not too far away from her own private attitude (i.e., she will want to be true to herself), but she will also tend to conform to the prevailing social norm. We refer to these tendencies as *authenticity preference* and *social extremeness aversion*, respectively, and assume that expressed attitudes will reflect a utility-maximizing compromise between these often-opposing principles.^4^

To illustrate, suppose Alison expresses her median political attitude of .3. This viewpoint of .3 would lie in the most liberal few percent of the social norm distribution that Alison has inferred from observation of her neighbors (see Figure 1C), and although her authenticity preference would be completely satisfied, Alison would lose utility due to being “socially extreme.” Alternatively, Alison could express an attitude of .75. She would then be conforming completely to the social norm, and would experience no social extremeness aversion, but her authenticity preference would not be well satisfied.

If Alison expresses the “compromise” attitude shown by the vertical dashed line (i.e., .48; see Figure 1D) the social extremeness of her expressed attitude will be less than if she expresses her “authentic” attitude of .3, but she will only partly satisfy her authenticity preference. This compromise attitude is close to the center of the political continuum and, therefore, represents an attitude that is moderate compared with the more extreme liberal position represented by Alison’s private attitude. We assume that Alison loses utility to the extent that this expressed attitude is extreme within the distribution that represents her private attitude. The extent of this loss is represented by the area shaded in dark gray in Figure 1E. The larger this area, the greater the loss of utility that is experienced. We also assume that Alison loses utility to the extent that this expressed attitude is extreme within the distribution that represents her perception of the social norm. The extent of this loss is represented by the area shaded in light gray in Figure 1F.

Below, we quantify authenticity preference and social extremeness aversion and show through simulation that in a range of conditions it is utility-maximizing for Alison to express a compromise attitude—that is, to allow her behavior to be affected by the social norm. We also explore conditions under which Alison’s behavior will be less affected by the social norm. For example, when the social norm is both highly homogeneous and located far away from Alison’s own attitude, Alison will lose much authenticity, yet still remain socially extreme, if she shifts her expressed attitude toward the social norm. Under such conditions it can be utility-maximizing for Alison to be “true to herself” and express her authentic attitude even in the face of an opposing social norm.

In a second set of simulations, we examine the behavior of a network of simulated agents in which each agent’s choice of attitude to express is governed by authenticity preference and social extremeness aversion as just described, and in which each agent’s social norm is determined by the expressed attitudes of its social network neighbors. Expressed attitudes therefore reflect the outcome of a complex dynamic interplay between network agents. Agents have an incentive to choose social network neighbors with attitudes similar to their own (this motivated behavior arises because the agents can then express attitudes that satisfy their authenticity preference without experiencing as much social extremeness aversion). Thus, if agents are allowed to change their social network locations, they exhibit homophily, and we show that this leads to polarization in the social network (polarization occurs when agents with extreme underlying private attitudes become less constrained in their choice of attitude to express after they change their social/informational environment to exclude the moderating influence of opposing attitudes).

Thus, two key assumptions in SST concern social extremeness aversion and authenticity preference. Both are independently motivated. The idea of extremeness aversion as we use it here originates in the literature on judgment and decision-making and captures the idea that people typically choose “compromise” options (Simonson & Tversky, 1992). Extremeness aversion has been shown to influence real-world consumer choices of, for example, food portion size (Sharpe et al., 2008); SST extends the notion of extremeness aversion to the domain of social norms. There is also a large body of research on social conformity in a variety of fields (e.g., Bernheim, 1994; Bicchieri, 2006; Claidière & Whiten, 2012); we touch on aspects of this literature throughout.

The authenticity preference assumption is intended to have intuitive plausibility, but also receives support from research on the relationship between subjective well-being and various conceptions of authenticity. One conceptualization of authenticity focuses on the consistency with which personality traits are expressed in different social contexts (e.g., Sheldon et al., 1997); another examines self-ratings of “falseness to self” and related constructs (Harter et al., 1996). The recent Tripartite Model of authenticity (Wood et al., 2008) has “accepting social influence” as one of its factors, and there are clear correlations between authenticity and various measures of subjective well-being. While such findings provide some motivation for our model, in that we assume utility is lost to the extent that overt behavior (expressed attitudes) departs from what would be mandated by internal private attitudes alone, we make no claim that our simple quantification captures the full richness of current psychological conceptions of authenticity.

The model aspires to offer some simple principles that apply to a wide range of phenomena rather than provide detailed fits to specific sets of data. Table 1 summarizes the “stylized facts” that we model below, and also shows parameter values for all reported simulations.[Table tbl1]

## Background Assumptions

Our cognitive approach to social comparison is motivated by and builds on three separate traditions of research. The first concerns the idea that people rely on small samples, drawn either from their memories or from the environment, in estimating quantities; the second has developed rank-based models of exactly how judgments are influenced by the context of comparison, and the third aims to integrate individual-cognitive and social levels of description through social simulation using agent-based models (ABMs). We briefly outline the background for each of these.

### Judgment and Inference Based on Small Samples

We assume that social judgments—such as an estimate of a social norm—are made on the basis of small samples retrieved from memory at the time the judgment must be made (see also Galesic et al., 2012, 2018; Pachur et al., 2013; Stewart et al., 2006). This approach is consistent with findings that judgments are often made on the basis of a relatively small number of observations that are either recalled or immediately available in an experimental environment at the time judgment must be made (Fiedler, 2000; Fiedler & Juslin, 2006; Juslin et al., 2007). The use of small samples may amplify differences between alternative payoffs (Hertwig & Pleskac, 2010) and correlations (Kareev, 1995, 2000), but can also lead to biased judgments (Fiedler & Juslin, 2006; Kareev, 2000). This bias, which can result from the polarization and segregation mechanisms that we describe below, is responsible for a number of the phenomena we aim to account for.

Following some other recent models (e.g., Galesic et al., 2012; Pachur et al., 2013), as well as older accounts from an agent-based modeling tradition (e.g., Latané & Wolf, 1981; Nowak et al., 1990) and mathematical sociology (e.g., Social Influence Network Theory: Friedkin & Johnsen, 2011), we assume that the process of sampling from the immediate (local) social environment may explain various social phenomena (see also Bergh & Lindskog, 2019; Denrell, 2005; Galesic et al., 2018; Hills & Pachur, 2012; Schulze et al., 2020).

Our research builds on and extends these perspectives by offering a utility-maximizing framework to explain exactly how it is that biased social or informational sampling links to individual perceptions of social norms.

### Rank-Based Relative Judgments

We have already mentioned SST’s assumption that a social norm is represented as a distribution rather than a single point. This assumption allows us to model the evaluation of an expressed attitude or attitude-related belief (e.g., for its social extremeness) not by how it relates to the average attitude expressed by others (a mean-based social norm) but instead by how it ranks in the distribution of others’ attitudes (a rank-based social norm).

The assumption that judgments are made relative to a single comparison point, which is typically some measure of the central tendency of contextual items (cf. Helson, 1964) and often simply taken to be the mean, is made in many areas of psychology. We refer to this as a *mean-relative* approach. Thus, it is often suggested that quantities such as prices, healthy body weights, or amounts of alcohol consumption are evaluated in terms of their relationship to a “reference,” “typical,” or “average” level (e.g., Blanchflower et al., 2009; Mazumdar et al., 2005). According to the mean-relative approach, a person might judge their alcohol consumption as excessive to the extent that it exceeds others’ average level of drinking (Neighbors et al., 2004), or they might compare their income with the mean income of an occupational or social reference group of some kind (e.g., Clark & Oswald, 1996). The same assumption of mean-relative judgment is often implicit in social norm interventions, where people are given information about mean energy consumption about the mean level of others’ energy usage (Schultz et al., 2007), alcohol consumption (Neighbors et al., 2004), or contributions to a public goods game (Fischbacher & Gächter, 2010) in the expectation that behavior will tend to adjust in the direction of the social norm (see also Lewin, 1952).

However, both intuition and experimental evidence call the mean-relative approach into question. Consider, for example, a person who is informed that they use 120 units of energy per month, while their social network neighbors use 90, 100, and 110 units per month. Intuitively, the person will feel that their consumption is rather high in relation to the social norm. Suppose that the same individual was instead told that their social network neighbors use 20, 100, and 180 units of energy per month. It seems likely that the person’s consumption of 120 units per month will feel subjectively less deviant from the social norm—less socially extreme—in this second context. But the mean of others’ consumptions is 100 in both cases, suggesting that people are sensitive to how their own behavior ranks within the distribution represented by the social norm.

The general idea that subjective judgments are often judgments of relative rank within some distribution was initially developed in the context of psychophysical judgment (Parducci, 1965, 1995), and findings of rank effects in that domain were subsequently extended to domains as diverse as, for example, sweetness perception (Riskey et al., 1979), moral judgments (Marsh & Parducci, 1978), perception of body image (Wedell et al., 2005), student grading fairness (Wedell et al., 1989), and prices (Niedrich et al., 2001, 2009). Judgments of “fair” allocations of wage and tax increases also follow rank-based principles (Mellers, 1982, 1986), as do judgments of other economic quantities (Boyce et al., 2010; Brown et al., 2008; Smith et al., 1989) and event-rated death tolls (Olivola & Sagara, 2009). In process terms, rank-based judgments may be formed through a process of sampling followed by binary ordinal comparison (Stewart et al., 2006) or by directly estimating value in a cumulative distribution; we return to the distinction between these in the General Discussion.

According to this rank-based perspective, people’s judgments should be affected by (a) their beliefs about social norm distributions, along with (b) their belief about where they rank within that distribution. Consistent with such a view, an individual’s belief about where their own behavior (e.g., their alcohol consumption or exercise levels) ranks within a perceived social norm (“subjective rank,” e.g., whether they are in the heaviest-drinking 15% of the population) predicts that individual’s attitude toward their own behavior (e.g., whether they are drinking “too much”). Judgments are based on people’s personal beliefs about their social rank not just for quantities such alcohol and exercise amount (Maltby et al., 2012; Taylor et al., 2015; Wood et al., 2012), but for quantities as varied as food healthiness and food consumption (Aldrovandi, Brown, et al., 2015), depression and anxiety symptoms (Melrose et al., 2013), student indebtedness (Aldrovandi, Wood, et al., 2015), dishonesty (Aldrovandi et al., 2013), and student experience (Brown et al., 2015).

In summary, there is considerable independent support for the idea that people’s judgments and attitudes are influenced by where they believe themselves to rank within a perceived distribution that represents a social norm. SST aims to provide a quantitative footing for the psychological processes underlying these rank-based social norm effects.

### Agent-Based Modeling Approaches

A further aim of SST is to show how rank-based social judgments at the individual level, as reviewed in the previous section, can lead to emergent phenomena such as polarization at a social network level. To do this, we use a simple ABM. ABMs enable the collective behavior and emergent properties of neighborhood-sensitive agents to be studied (Easley & Kleinberg, 2010; Newman et al., 2002; Schelling, 1971) and have a long history of application in a number of social as well as physical sciences, with a particular focus having been on how group-level structure may emerge as a result of the actions of individuals. They have provided useful insights into areas such as collective behavior in ants and traders (Kirman, 1993), swarming behavior (Reynolds, 1987), crowd behavior (Dyer et al., 2009), population group size (Axtell et al., 2002), cultural dissemination (Axelrod, 1997), segregation (e.g., Schelling, 1978), and imitative voting (Bernardes et al., 2002). Within psychology, ABMs have been applied to the development of cooperation and the spread of behaviors through populations (see Goldstone & Janssen, 2005; Jackson et al., 2017; Macy & Willer, 2002; Madsen et al., 2019, for reviews from the perspectives of cognitive science, computational sociology, and social psychology).

Here, we exploit the ability of ABMs to bridge cognitive and social approaches, with the specific aim of showing how polarization can emerge from the tension between extremeness aversion (defined in terms of rank-based social norms) and authenticity preference. SST has a number of important predecessors, such as the cultural spread model (Axelrod, 1997), in which agents are endowed with beliefs and attitudes and interact with other agents with a probability that depends upon their overall similarity. Upon interaction, individuals become more similar to one another. There are numerous extant ABMs of, for example, segregation (e.g., Schelling, 1971), social impact (Nowak et al., 1990), and social influence (e.g., Bentley, Ormerod, et al., 2011; Flache & Macy, 2011a; Friedkin & Johnsen, 2011), and such models can shed light on social sampling and the biases that result from it (Galesic et al., 2012, 2018; Pachur et al., 2013; Schulze et al., 2020). Models of attitude and belief polarization have been developed within both ABM and Bayesian traditions (see especially Baldassarri & Bearman, 2007; Flache & Macy, 2011b; Jern et al., 2014; Mäs & Flache, 2013; Van Overwalle & Heylighen, 2006). Voinea (2016) provides a historical overview of simulation modeling approaches to political attitudes. SST differs from these earlier models particularly in (a) its emphasis on rank-based relative judgment, and (b) its focus on a tension between social extremeness aversion on the one hand and authenticity preference on the other. It is to these that we now turn.

## Model Overview

### Network Structure

**Figure 2 fig2:**
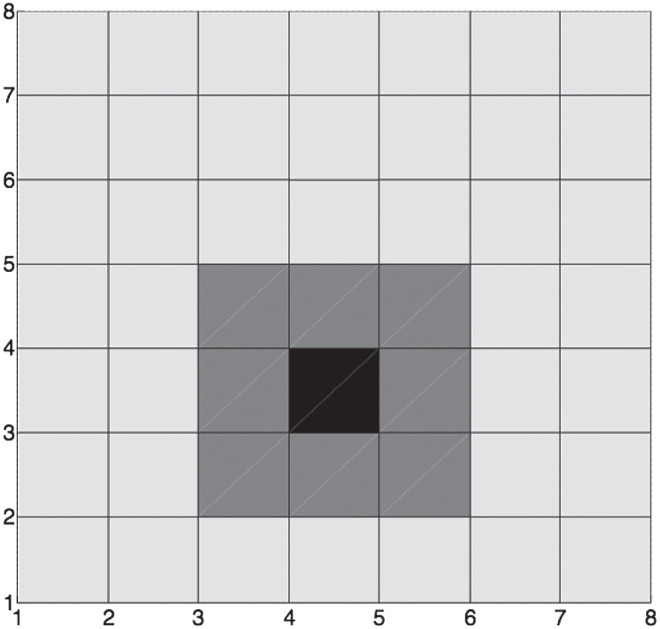
Social Neighborhood (Gray Squares) of an Agent (Dark Square) Within a Larger Neighborhood (Light Squares)

We model individual agents situated within a network. Each simulated social agent in the model occupies a location on the grid, and can observe the behavior only of its eight local neighbors. This neighborhood structure is illustrated in Figure 2, where the black square highlights one agent in a 7 × 7 grid and the gray squares highlight the immediate neighbors whose behaviors (i.e., expressions of attitudes) are visible to that agent.[Fig fig2]

Although the network of agents is modeled (and graphically illustrated) as a spatial grid, the grid dimensions can represent any social or informational dimensions relevant to a social norm. For example, the grid could be taken to represent a social structure such that agents occupying a particular location in the grid spend social time with agents in nearby locations (which need not be physical locations but could be socially constructed “locations” such as shared blogs). The network structure can therefore be thought of as informational (surrounding network locations represent sources of information, such as blogs or newspapers, that the agent attends to); we adopt a simple spatial interpretation for the purposes of explanation.

In the simulations below, we explore the effects of allowing agents to move to different locations in the network; this represents the agent choosing to associate with other agents located in a particular area of the grid (e.g., because those agents share similar political or other views to those of the agents who move) or attending to particular sources of information (e.g., newspapers). In other words, a move of an agent from one location to another will in reality more often represent a choice to socialize with, read the same newspapers as, and influence and be influenced by, a particular set of agents rather than a decision to move house to a different region of the city.

The neighborhood is a torus—that is, it wraps round along horizontal and vertical edges. Thus, an agent in a given row in the extreme right-hand column of grid will have as one of its neighbors the agent in the same row of the extreme left-hand column of the grid. In most of the simulations described below, we use a 100 × 100 grid. The simple network structure shown here is similar to that introduced by, for example, Schelling (1969, 1971) to illustrate how neighborhood segregation could occur as a result of people having even slight preferences to move to a location where they were surrounded by same-race neighbors, except that we do not allow empty locations. The Schelling approach has sparked the development of an enormous number of derivative versions and applications in disparate disciplines (see, e.g., Rogers & McKane, 2011, for a recent analysis); we do not review these here.

### Agents’ Private Attitudes

Each agent *i* in the network is endowed with a fixed and private attitude defined as a distribution over an interval between 0 and 1 on the dimension of interest. For example, as in the informal example discussed earlier, the number between 0 and 1 could represent a one-dimensional political attitude, where 0 represents an extreme left-wing attitude and 1 represents an extreme right-wing attitude. We will use this example of political attitude to illustrate many of the points below. However, the same type of representation is assumed to underpin attitudes more generally.

Each agent’s underlying private attitude is assigned randomly at the outset and remains fixed throughout most the simulations reported here. Our account thus assumes private attitudes to be fixed characteristics of individuals, akin to deeply held values or personality traits, which typically show strong evidence of both heritability and stability over the lifespan (see, e.g., Mondak, 2010). In Demonstration S2 (in Supplementary Online Material), we do however explore the consequences of relaxing this assumption, and show that allowing private attitudes to move incrementally in the direction of expressed attitudes leads to reducing, rather than increasing, polarization over time.

A central assumption of our model is that agents’ private attitudes are not directly observable by social network neighbors. Instead, social network neighbors have access only to the overt behavior (e.g., behavioral statements of attitudes) of other agents. This overt behavior is, as we explain below, assumed to be influenced by additional factors, such as social norms, which do not influence the private underlying attitudes.

Specifically, we assume that each agent’s private attitude along a particular dimension can be expressed as a beta distribution. A beta distribution is bounded between 0 and 1 and has two shape parameters, α and β, which together specify both the central tendency of the distribution and its width (variance). Each individual *i* in the social network has their own α and β parameters, α_*i*_ and β_*i*_, and hence their private attitude is given by beta (α_*i*_, β_*i*_).

**Figure 3 fig3:**
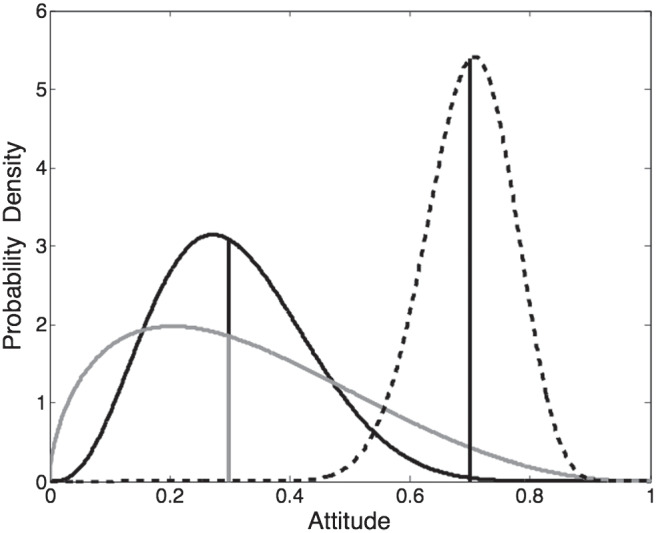
Attitudes as Distributions *Note*. Illustration of attitudes with different medians (.3 for light- and dark-solid lines; .7 for dashed line) and different precisions but the same median (higher precision for dark-solid line than light-solid line)

Representing attitudes as distributions rather than single points allows us to distinguish between the central tendency of the distribution and the strength with which it is held.^5^ In intuitive terms, there is a distinction between “extreme views weakly held” and “moderate views strongly held” that can be captured only if the central tendency and variance of the attitude are separately represented. Figure 3 illustrates the representation of different attitudes. The black-solid curve is beta (4, 9) and represents an attitude with a central tendency (median) of .3 held with medium strength. Using the example of political attitude, this could represent a slightly left of center (liberal) attitude held with a moderate degree of conviction.[Fig fig3]

We define the width of the distribution in terms of its precision (i.e., the reciprocal of the variance); in this case the precision is 65.7. As α and β become smaller, the distribution becomes shallower, representing a less strongly held attitude. The gray-solid line shows beta (1.6, 3.3); this is constructed to have the same median (.3), but the precision is reduced to 27.0. To continue the example, this could represent the same left of center (liberal) attitude held with a low degree of conviction. Finally, the dashed curve shows beta (27, 11.8); this would represent a moderately conservative political attitude (*mdn* = .7) held with a high degree of conviction (precision = 188). The two shape parameters of the beta distribution, α and β, can be given a psychological interpretation in that they can be thought of as representing the number of arguments considered by the agent to favor one or other end of the attitude dimension (cf. Koriat, 2012).

The private attitudes that we have described are assumed in our model to be an important, but not the only, influence on actual behavior. Next, we explain how social norms are estimated in the model and how they, combined with private attitudes, influence agents’ choices of what attitude to express.

### Inferring Social Norms

Recall that each agent is assumed to have access through observation to the expressed attitudes of its eight local neighbors (Figure 2). The second assumption of the model is that agents represent social norms as distributions, and that they infer these social norms from observing the expressed attitudes of their network neighbors, as illustrated in Figure 1. We label the parameters of the social norm that describes the neighbors of agent *i* as α_*ni*_ and β_*ni*_, and hence the social norm is given by beta (α_*ni*_, β_*ni*_).

As with private attitudes, representing social norms by distributions rather than single points allows the central tendency of a social norm to be represented independently of the degree of social consensus surrounding it. The attitudes expressed by Alison’s neighbors could have been more homogeneous (e.g., clustered much more tightly around a median of .7), in which case Alison’s representation of the social norm would be taller and narrower. Alternatively, a shallower (lower consensus) social norm with the same median could be inferred if the attitudes expressed by Alison’s neighbors were more heterogeneous. As we will see below, the ability to represent the degree of social consensus in this way will be important in understanding the magnitude and even the direction of social norm influences.

### Authenticity Preference

It is assumed that—absent other considerations—an individual will prefer to express an attitude near the center (here operationalized as the median) of the distribution that represents their own private attitude. To the extent that their expressed attitudes depart from their true attitudes in order to accommodate other constraints such as the desire not to occupy an extreme location within the social norm (see below), they lose utility. This is the *authenticity preference* assumption. The degree to which an expressed attitude departs from this median will be determined by how much the relative ranked position of the expressed attitude (within the cumulative of the distribution that represents the authentic attitude) departs from .5. Formally, if the expressed attitude is denoted by *A*_*i*_ and *A*_*i*_ is greater than the median of the private attitude, the utility loss increases with

$$I_{A_{i}}(\alpha_{i},\beta_{i})-0.5,$$ 1

which is the dark-shaded area in Figure 1E. *I*_*x*_ (α, β) represents the position of *x* in the cumulative density function of beta (α, β), and *I*_*x*_^[−1]^(α, β) represents the inverse of the cumulative density function. Thus, the preference for authenticity would be met completely only when *A*_*i*_ is the median of the private attitude, that is, when *A*_*i*_ = *I*_.5_^[−1]^(α_*i*_, β_*i*_).

To give a concrete example, recall that Figure 3 illustrates two different authentic attitudes—both with the same median (.3) but differing in precision. Consider the loss of authenticity that will result from expressing an attitude of .4 in each case. The relative ranked position of .4 in the more precise distribution (i.e., its position in the cumulative density function) is .77, and hence (by Equation 1 above) the loss of utility associated with expressing the .4 will be .77−.5 = .27. In contrast, the relative ranked position of .4 in the less precise distribution is .67, and the associated utility loss would be .17. Thus, the loss of authenticity associated with expressing an attitude that is a fixed distance (here, .1) from the central tendency of the authentic attitude is greater when the authentic attitude is more precise.

In other words, the loss of utility associated with the preference for authenticity does not depend just on the distance between the (median) private attitude and the expressed attitude *A*_*i*_. Instead, the loss of utility will depend also on the width of the private attitude distribution. If the distribution is narrow, indicating that precision is high, a shift in attitude of .1 away from the median attitude will cause a greater loss of utility than will the same shift in attitude when the private attitude distribution is less precise. This property captures the intuition that it is more painful to express behavior that is inconsistent with a strongly held attitude than it is to express the same behavior in the context of a less strongly held attitude. This intuition cannot be captured by single-point representations of attitudes.

### Social Extremeness Aversion

We define social extremeness aversion as extremity (distance from the median) in the rank ordered distribution that represents the social norm. Specifically, we assume that the loss of utility arising from social extremeness aversion is given by the probability mass in the social norm distribution that separates the expressed attitude from the median of the social norm. This is shown as the light-gray area in Figure 1F. Formally, the utility loss arising from social extremity is an increasing function of

$$0.5-I(\alpha_{ni},\beta_{ni}),$$ 2

where *I* (α, β) is again the cumulative beta distribution function.^6^ As with authenticity preference, an important feature of this formulation is that disutility arises not simply from the distance between an expressed behavior (.48 in the Alison example) and a single-point estimate of the social norm (we take the median, here .7). Instead, and in contrast to most extant models of social norms, what is assumed to matter is the relative ranked position of an expressed attitude within the distribution that represents the social norm. The disutility will therefore depend on the degree of social consensus that underpins the social norm. If there is a high degree of social consensus, the probability distribution that represents the social norm will be sharply peaked and a difference of .22 between the median of the social norm and the expressed attitude will lead to a greater loss of utility than would be produced by the same .22 difference if the social norm is wider (as it will be if there is a lower degree of social consensus). This way of implementing social extremeness aversion captures the intuition that loss of utility derived from expressing a view that is different from the median view expressed within a social neighborhood will be greater when neighbors are in agreement than when they are not, and provides another illustration of the importance of representing norms as distributions rather than single points.

### Choice of Behavior

How do the agents choose what attitude and behavior to express in the light of the twin constraints of authenticity preference and social extremeness aversion? We assume the agents are rational in the sense that the attitude *A*_*i*_ that each chooses to express in a given social context is the one that will maximize their utility. In cases such as those we have illustrated, the maximum-utility choice of expressed attitude behavior will fall somewhere between the median of the agent’s private attitude and the median of the social norm distribution.

**Figure 4 fig4:**
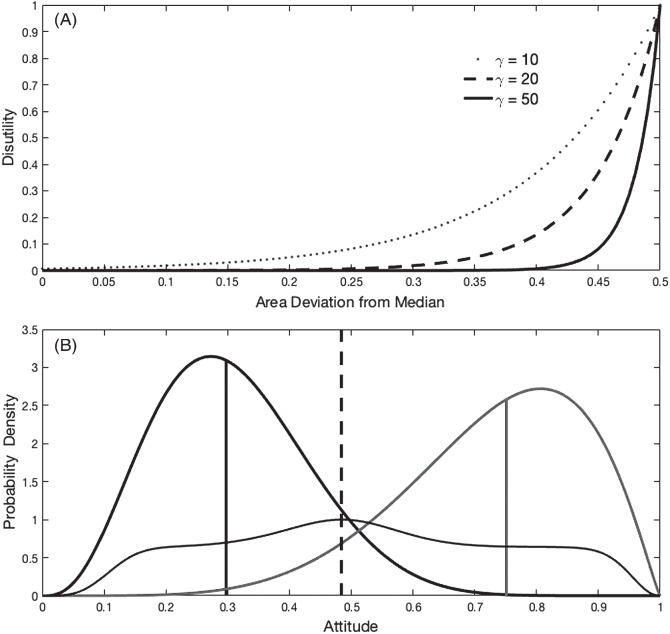
Loss of Utility when Attitudes are Socially Extreme or Inauthentic *Note*. 4A: Disutility as a function of departure from authenticity and social norm. 4B: Utility (thin-dark line) as a function of expressed attitude (horizontal axis) given the private attitude illustrated by the thicker dark line and the social norm given by the lighter solid line.

We have already shown that authenticity preference and social extremeness aversion will often tend to pull *A*_*i*_ in opposite directions. We have stated that utility loss increases as a function of both the dark-shaded and light-shaded areas in Figure 1F. Our final assumption concerns the rate at which disutility increases as each of those areas increases. We aimed to instantiate the intuition that utility loss will be relatively small when departures from the median are relatively small, but then will increase sharply as extremeness increases. Specifically, we assume that this disutility increases as an exponential function of each of the areas illustrated, such that

$$\text{Disutility}=e^{\gamma \left(H-.5\right)},$$ 3

where *H* is the relevant area and γ, the first free parameter of the model, specifies the steepness of the increase. Figure 4A shows this function for three different values of γ (10, 20, and 50). Consider the parameter that characterizes the middle of the three lines (i.e., γ = 20). With this parameter value, there would be little or no loss of utility due to social extremeness aversion provided the expressed attitude *A*_*i*_ lies within about the 25th and 50th percentile of the social norm. As social extremity increases, however, the loss of utility increases ever more quickly such that there is a considerable loss of utility if the expressed attitude lies within the most extreme 5% or 10% of the social norm distribution. The same equation applies to authenticity preference; here, the intuition is that one is comfortable to express an attitude that falls within the middle two quartiles of the distribution that represents one’s private attitude, but then increasingly loses utility with diminishing authenticity such that considerable well-being will be lost if an attitude at the extreme of the private attitude distribution must be expressed. In Demonstration S1 in the Supplementary Online Material, we show that this assumption (of increasingly large increases in disutility at extremes) is consistent with data from social norm effects in a resource dilemma experiment (Bilderbeck et al., 2014).[Fig fig4]

Combining Equations 1, 2, and 3 above, the overall (positive) utility associated with the expression of a given attitude *A*_*i*_ will be,

$$U_{A_{i}}=1-[w\times e^{-\gamma (I_{A_{i}}\left(\alpha_{ni},\beta_{ni}\right)}+(1-w)\times e^{-\gamma \left(1-I_{A_{i}}\left(\alpha_{i},\beta_{i}\right)\right)}],$$ 4

where *w* is a weighting term, here set to .5 by default, such that *w* is the weight on the loss of utility due to social extremeness aversion and (1 − *w*) weights the loss of utility arising from the departure from authenticity preference.

Figure 4B plots the utility function (thin line) for the private attitude and social norm illustrated in Figure 1, with *w* = .5 and γ = 20. It can be seen that the utility-maximizing *A*_*i*_ is .48, at the point where the vertical-dashed line was (intentionally, in anticipation) drawn. As *w* becomes smaller than .5, the weighting on social extremeness aversion will reduce and the utility-maximizing *A*_*i*_ will become closer to the median of the agent’s private attitude.

Most of the psychologically interesting behavior of the model arises from the interplay of private attitudes and perceived social norms in determining agents’ utility-maximizing attitudes to express, and it is to this interplay that we now turn. We first explore how the utility-maximizing attitude for an individual agent to express is governed by the interaction between the precision of the agent’s own private attitude and the agent’s immediate social environment, then in a separate section explore how polarization and other network phenomena can emerge from simple network dynamics.

## Individual Agent Simulations

In this first series of simulations, we examine how the behavior of a single agent is influenced by interactions between (a) the strength of the agent’s private/authentic attitude and (b) the degree of consensus underlying the social norm.

### Social Norm Effects

Numerous studies both in the laboratory and in the field have examined the effects of telling people what others do or believe. Provision of social norm information can, for example, influence provision of movie ratings (Chen et al., 2010), size of voluntary gallery donations (Martin & Randal, 2010), and energy consumption (Allcott & Rogers, 2014; Ayres et al., 2013; Schultz et al., 2007), as well as contributions to public goods in economic games (Fischbacher & Gächter, 2010) and preferences for music (Salganik et al., 2006). There are many accounts of why descriptive and other social norms might have the effects that they do (see, e.g., Morris et al., 2015); the focus here is specifically on the psychological processes that underlie the influence of social norms on the attitudes that people express.

#### Demonstration 1.1: Effects of Social Comparison on Expressed Attitudes and Well-Being

We first illustrate the effects of individual differences in sensitivity to social norms to provide a quantitative illustration of the effects of authenticity preference.

Intuition suggests that some people have a strong tendency to conform to a social norm, while others are more likely to stay “true to themselves” and are less likely to alter the views they express when they are around others who are expressing different views. In SST, this individual difference is captured by the parameter *w* (Equation 4), which specifies the weight that an agent gives to disutility arising from social extremeness aversion relative to the weight given to authenticity preference (i.e., a value of *w* = 0 would describe an agent who has the strongest possible authenticity preference).

We examine the effects of varying *w* on the expressed attitude of an agent whose true attitude is given by beta (4,9) as illustrated in Figure 3 above—that is, the median of the attitude is 0.3 (e.g., slightly to the left of the political center) and the attitude is held with moderate strength (the distribution is neither very sharply peaked nor very flat). The agent’s neighbors are assumed to have the distribution beta (6, 2.2; i.e., there is a moderate level of agreement between social neighbors on a politically right-wing attitude such that the distribution representing the social norm is neither sharply peaked nor very flat). The parameter γ is held fixed at 20.

**Figure 5 fig5:**
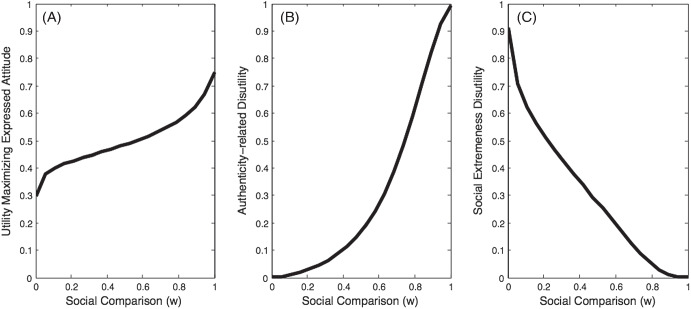
Effect of Varying w Parameter (Relative Concern With Social Extremeness Aversion) *Note*. Panel A: Utility-maximizing attitude as a function of *w*. Panel B: Disutility due to violation of authenticity preference. Panel C: Reduction in disutility due to social extremeness aversion

Figure 5A shows how the utility-maximizing attitude for the agent to express changes as a function of *w*. As the social comparison parameter *w* increases, the agent’s expressed attitude (i.e., the one whose expression maximizes the agent’s utility) gradually moves away from the agent’s median authentic attitude (.3) toward the median of the social norm (.75) and the amount of disutility due to violation of authenticity preference increases (Figure 5B). The concomitant reduction in disutility due to social extremeness aversion is shown in Figure 5C.[Fig fig5]

#### Demonstration 1.2: Effects of Social Norm Consensus

**Figure 6 fig6:**
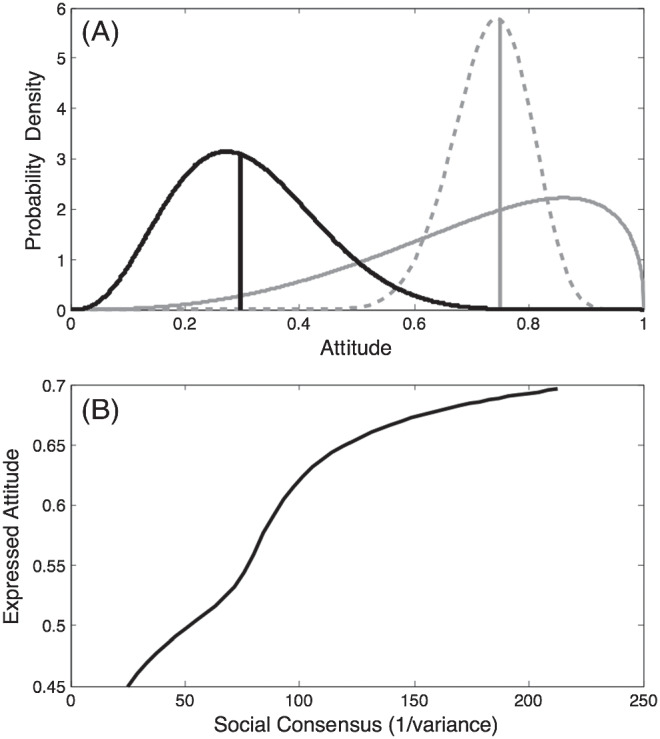
Effect of Social Consensus on Expressed Attitudes *Note*. Top panel: Agent’s private attitude (dark line) and high consensus (dotted line) or low consensus (gray line) social norms. Bottom panel: Effect of varying social consensus

SST aims to capture, quantitatively, the idea that one is likely to be more influenced in the statement one expresses when there is a higher degree of social consensus surrounding an opposing viewpoint (cf. Asch, 1956). We illustrate the effect of social consensus in Figure 6. The private attitude of the agent, as in the previous simulation, is given by beta (4,9), has a median of .3, and is illustrated as a probability distribution (dark line) in the top panel. However, we vary the social norm from low consensus, beta (3.6, 1.4); solid-gray line, to high consensus, beta (30, 10.2); dashed-gray line, while holding the median (almost exactly) constant at .75. We measure the degree of social consensus as the reciprocal of the variance (i.e., the precision) of the distribution representing the social norm. The social comparison parameter *w* is held constant at .5.[Fig fig6]

The effect of increasing social consensus on an agent’s utility-maximizing *A*_*i*_ is illustrated in the lower panel of Figure 6. As the variance in the social norm decreases, the expressed attitude that maximizes utility for the agent gradually moves from a compromise position of .45 to become ever closer to the median of the social norm. The intuition behind this result is as follows. If the views expressed by neighbors are highly diverse (high variance/low precision), the social norm distribution will be relatively flat. In such a case, when choosing the optimal attitude to express an agent does not have to move very far from its own true authentic belief in order to avoid being too socially extreme. Because an agent always wants to express a belief as close as possible to its true authentic belief, it will do so provided the cost in social extremeness is not too high. As the distribution that represents the social norm becomes narrower, the agent comes to express a view that is further away from its own authentic attitude in order to avoid being too extreme in the social distribution.

This demonstration highlights a key difference between SST and alternative accounts based on single-point representations of attitudes and social norms. In a model with single-point representations, the disutility of an agent could be a function simply of the distance between the single-point social norm and the agent’s expressed attitude, perhaps along with the distance between the agent’s authentic preference and the agent’s expressed attitude. The difference between the two representations is relevant to which type of social norm information would be most effective. Norm-based “nudges” typically report the mean of the relevant distribution (e.g., alcohol consumption or energy usage). However, if SST’s assumption about social extremeness aversion is correct, it may be more effective to tell people where they rank within a social distribution than to tell them how they relate to the mean of a distribution.

#### Demonstration 1.3: Backfire Effects

The principles of extremeness aversion and authenticity preference, coupled with the assumption that private attitudes and social norms are represented psychologically as distributions rather than single points, can lead to otherwise-paradoxical non-monotonic effects of social norms on expressed attitudes. One of these is the so-called backfire (or boomerang) effect.

Backfire effects are typically said to occur when the provision of new information that is inconsistent with an existing belief or attitude may under some circumstances paradoxically lead to further entrenchment of the original opinion. Backfire effects have been seen when the relevant new information takes the form of empirical facts (Bail et al., 2018; Gollust et al., 2009; Nyhan et al., 2013; Nyhan & Reifler, 2010; Redlawsk, 2002) or when the information is in the form of a social norm. For example, Hart and Nisbet (2012) found that Republicans became less, not more, supportive of climate mitigation policies when provided with information about the potential health impacts of climate change. Costa and Kahn (2013) find that provision of social norm information related to energy conservation may backfire with conservatives despite being successful with liberals, and Cook and Lewandowsky (2016) found that providing information about scientific consensus (about anthropogenic global warming) led to backfire effects specifically in strong supporters of unregulated free markets.

Backfire effects are not always found (e.g., Guess & Coppock, 2018; Wood & Porter, 2019), perhaps reflecting differences in tested populations (Wood & Porter, 2019), whether or not the new information is general enough to threaten pre-existing attitudes or beliefs (Ecker & Ang, 2019), or perceptions of the reasons for the consensus that may provoke the reaction (Conway & Schaller, 2005). Here, we examine the conditions under which backfire effects occur in SST. We set *w* to .3, instead of the .5 used in the previous demonstration, and again examined the optimal attitude for an agent to express as the social consensus increases—just as in the previous demonstration, and with the social norm varying continuously from beta (2.4,1.0; low social consensus) to beta (21,7.2; high social consensus).

**Figure 7 fig7:**
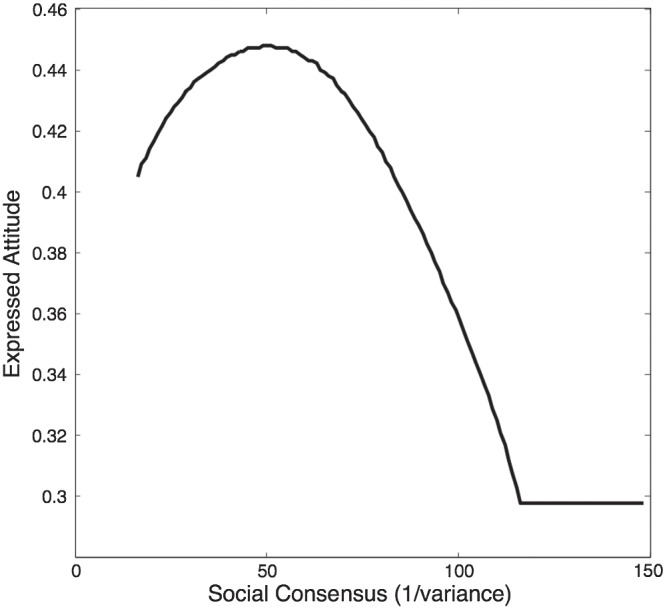
A Backfire Effect: Expressed Attitude as a Function of Social Consensus

The result can be seen in Figure 7, where the pattern that is observed is very different to the monotonic effect of social consensus observed in the previous demonstration. Instead, a backfire effect is observed. As the social consensus increases, there is an initial tendency for the agent’s expressed attitude to follow the social norm. However, when social consensus (expressed in terms of precision) reaches about 60, the expressed attitude that is utility-maximizing for the agent stops moving toward the social norm and gradually moves back toward the median of the agent’s private attitude, eventually reaching it when social consensus has reached a level of about 120. Thus, the model exhibits a backfire effect; there comes a point where increased perceived social consensus (which could arise, e.g., if an increasing number of social network neighbors were observed to express a similar attitude to the one expressed by already-sampled network neighbors) leads to reduced instead of increased conformity.[Fig fig7]

This apparently paradoxical behavior of the model can be understood as follows. In intuitive terms, when the agent’s private attitude is very distant from the social norm there comes a point where an agent is better off being “true to itself” than conforming even a small amount. More specifically: When the social consensus is high and the median of the social consensus is located far from an agent’s private attitude, the median of the private attitude will be out on the flat tail of the social norm. Under such circumstances even a small movement away from the agent’s true authentic belief causes the normal loss of authenticity-related utility while achieving little gain in conformity-related utility, because the agent is so far out in the tail of the social norm distribution that virtually no reduction in social extremism can be achieved by movement toward the social norm.

**Figure 8 fig8:**
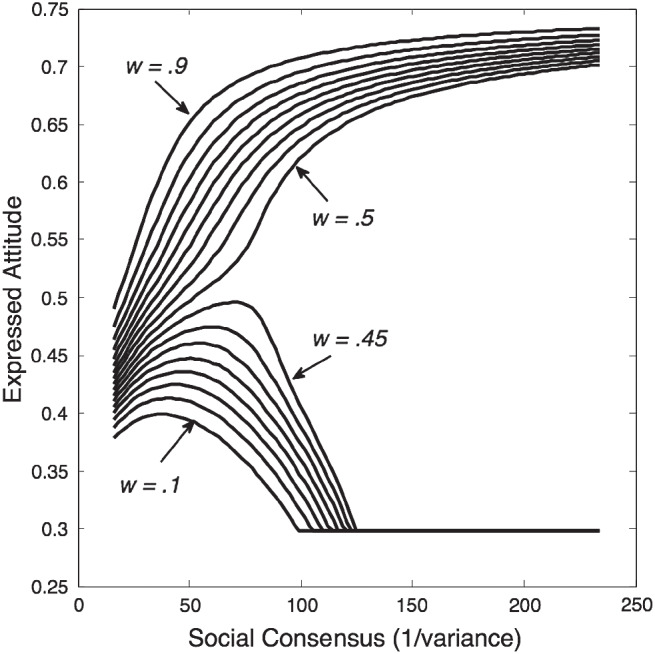
Backfire Effects as a Function of Social Consensus Effects and Parameter w

We can use SST to explore further the conditions under which backfire effects occur. Authenticity preference and social extremeness aversion and SST’s free parameters—*w* (which determines the amount of social comparison) and γ—will interact with the precision of agents’ private attitudes and of the social norm, as well as with the distance between them, to determine whether backfire effects occur. For example, Figure 8 shows the effect of varying *w* (all other parameters are the same as in the previous demonstration). It can be seen that for small values of *w*, backfire effects occur, but that as *w* increases there is a sudden transition to an environment in which conformity increases monotonically with social consensus.[Fig fig8]

**Figure 9 fig9:**
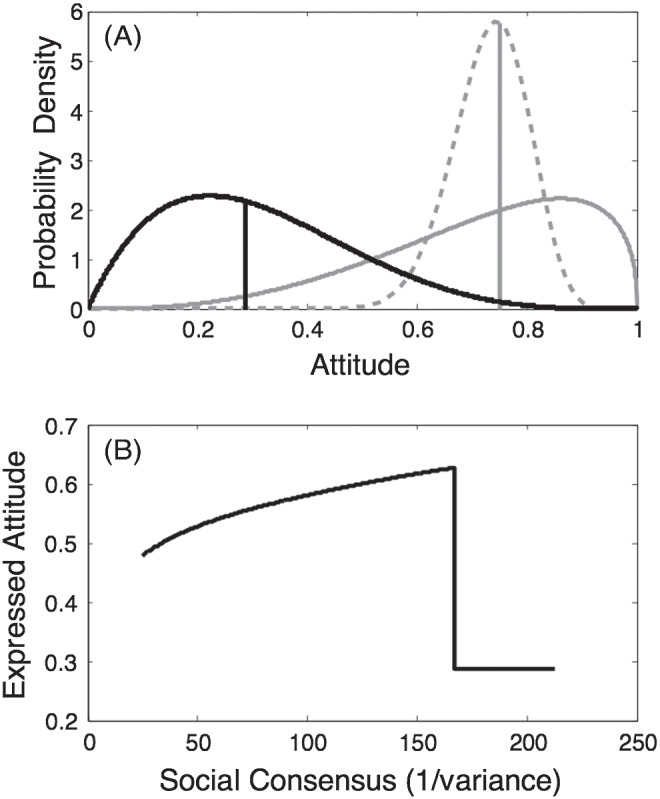
Discontinuous Change in Expressed Attitude as a Function of Social Consensus

The precision of an agent’s own world view is also important and either strong or weak private attitudes can lead to rapid step changes in expressed attitudes as social consensus increases. Figure 9 presents one such case: Here, the precision of the private attitude has been reduced to beta (2,4.5) (cf. Figure 6), but all other parameters remain the same as above (*w* = .3; γ = 20). As before, we examine the effect of increasing social consensus on the attitudes that it is optimal for the agent to express. Now there is a sudden switch in expressed attitude: At first the expressed attitude moves toward the social norm as consensus increases, but then there is a sudden change toward authenticity-maximizing behavior at a certain level of social consensus. The switch can arise because the utility curve is double peaked; the heights of the two peaks (whose locations correspond roughly to the authenticity-maximizing attitude on the one hand and the social extremeness-minimizing attitude on the other hand) change gradually (one increasing; the other decreasing) as consensus changes. There comes a point when the “authenticity peak” becomes higher than the “conformity peak,” and at that point there is a sudden change in the optimal attitude to express. In psychological terms, the model captures the intuition that there are conditions under which some social conformity is the most comfortable position for an agent, but that there are other conditions under which the utility-maximizing position is one of complete authenticity or complete conformity. Similar sudden transitions can occur in the opposite direction under different parameterizations.[Fig fig9]

## Simulations: Network Dynamics and Polarization

The demonstrations above illustrate how the behavior of utility-maximizing individual agents changes as a function of their private attitude, their perception of the social norm, and the *w* and γ parameters. The remaining demonstrations examine the network-level behavior of such agents with a particular emphasis on polarization, homophily, and social contagion. SST aims to offer a unified account of these phenomena, so we review them briefly together.

### Polarization

**Figure 10 fig10:**
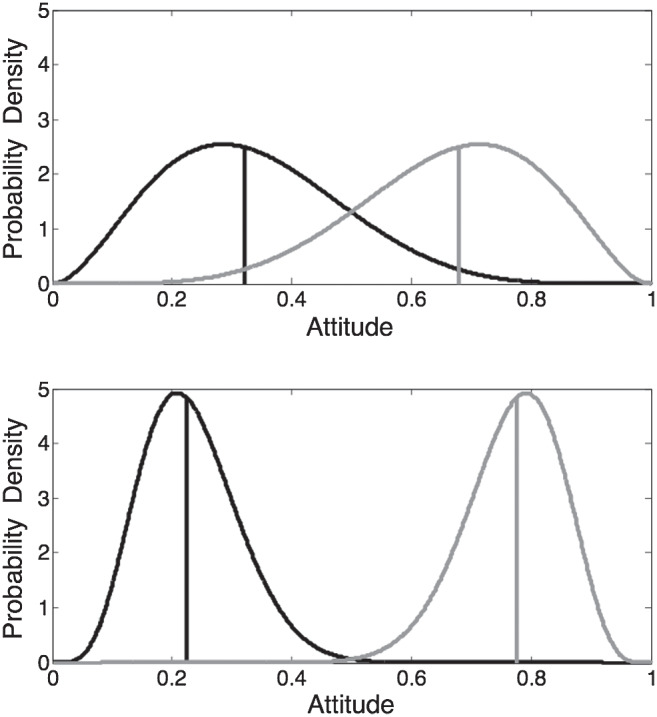
Stylized Illustration of Polarization

“Polarization” has been interpreted in a number of different ways (Bramson et al., 2017), but as we model it here is exemplified by the tendency for the attitudes and attitude-related beliefs expressed by a group and its members to be more extreme after within-group communication than before (Lord et al., 1979; Schkade et al., 2007). We illustrate the basic “stylized fact” in Figure 10. The horizontal axis represents endorsements of attitude-related statements (e.g., regarding contentious issues such as same-sex unions or the causes of global warming) on a 0–1 scale. The two distributions in the upper panel represent the distributions of expressed attitudes of two groups before discussion, while the two distributions in the lower panel represent the changed distributions of the attitudes of the two groups after each has had an opportunity for intragroup discussion. The distributions have become more homogeneous within groups, but more heterogeneous between groups—polarization has occurred. Although there are many subtleties in the large polarization literature, and perfectly clean patterns of the type illustrated may not be observed (see Sunstein, 2009 for a review) the figure illustrates the type of polarization that may be observed in small groups over short time scales (Schkade et al., 2007) and which we address with SST below (albeit in the context of a single group). Similar polarization is sometimes seen over longer timescales, as in the increased polarization evident in politicians’ voting patterns over recent decades (McCarty et al., 2006). We emphasize that our account focusses on attitudes rather than beliefs; the phenomena we aim to account for are different from (albeit closely related to) those that form the focus of models of *belief* polarization (e.g., Cook & Lewandowsky, 2016), whereby two individuals with initially opposing beliefs may have the difference between their beliefs strengthened by exposure to the same evidence (see Jern et al., 2014, for a review and model).[Fig fig10]

### Homophily and Confirmation Bias

Confirmation bias is the tendency to seek out information consistent with one’s existing beliefs or attitudes (Nickerson, 1998), with an associated tendency to maintain belief in existing hypotheses despite evidence to the contrary (Klayman & Ha, 1987). Confirmatory behavior may be adaptive if the task is to maximize information gain (see Navarro & Perfors, 2011; Oaksford & Chater, 1994), and may reflect a preference for belief consonance (Golman et al., 2017), but is typically seen as a bias and a contributor to polarization (Taber & Lodge, 2006). Here, we view the tendency to seek out others with attitudes similar to one’s own as a type of confirmation bias, and argue that SST provides a possible psychological mechanism for the bias. A related concept, social homophily, is the tendency for “birds of a feather to flock together”—that is, for people to seek out the company of similar others (McPherson et al., 2001). Our concern here is with what Lazarsfeld and Merton (1954) termed “value homophily” (a desire to surround oneself with people holding similar values) rather than with their “status homophily” (a desire to surround oneself with people occupying similar social groups).

Confirmation bias and homophily, and their consequences, are widely documented in the literature and numerous accounts have been proposed. Many of these existing accounts are similar in flavor to our own; the contribution of SST is to specify in detail the rank-based process of relative judgment at the level of the individual agents and how such a process can lead to confirmation bias and social organization at the network level. Specifically, SST proposes that confirmation bias and homophily can both be modeled by a simple trade-off involving two principal drives: individuals seek to maximize their authenticity-related utility while at the same time minimizing their disutility due to social extremeness aversion.

### Social Contagion Effects

Social contagion effects are said to occur when imitative behavior can be seen evolving in social networks over time, such that behaviors such as exercise, obesogenic eating, and high levels of alcohol consumption can be seen to propagate through social networks (e.g., Aral & Nicolaides, 2017; Christakis & Fowler, 2007, 2008; Datar & Nicosia, 2018), reflecting either social environments or the influence of one or more “leaders” (Dyer et al., 2009; Johnstone & Manica, 2011). Although interpretation remains controversial (e.g., Shalizi & Thomas, 2011), social contagion is often assumed to result from imitative behavior that does not merely reflect the tendency for people to surround themselves with similar others (homophily) or the tendency for neighboring social agents to be subject to common exogenous influences. We show below how social contagion effects can arise in SST as a result of social extremeness aversion even in the absence of homophily, but that the two are intertwined.

### Simulations

#### Demonstration 2.1: Polarization

We illustrate polarization with a simulation in which agents can choose to move to a different part of the network (thus changing their social/informational neighborhood) if they can increase their utility by so doing. In intuitive terms, polarization can occur because agents can typically increase their utility by moving to parts of the network where the agents have similar attitudes to their own. This utility increase occurs because inhabiting a more congenial neighborhood allows agents to express behaviors that are closer to their own private attitudes—thereby gaining authenticity—without suffering too much disutility from social extremeness.^7^

For example, consider again relatively liberal Alison (Figure 1). It is evident from the expressed attitudes of her network neighbors that she inhabits a fairly conservative neighborhood. As a result of her location, and of her social extremeness aversion, she loses utility because her authenticity preference cannot be well satisfied, if she is to avoid being socially extreme. Suppose that, in a different and more liberal local neighborhood within another part of the network, Alison has a Republican counterpart—Adam. Adam’s situation is the complement of Alison’s; assuming that Adam is also sensitive to social extremeness, he will be expressing a more moderate viewpoint in his liberal milieu than he would if he cared only about his authenticity preference, and is thereby losing authenticity-related utility. Imagine however how the situation would change if Adam and Alison were able to exchange network locations. The utility of both of them would improve. Alison’s utility-maximizing *A*_*i*_ will move to the left, because she can now express something more consistent with her private attitude without being so socially extreme. Her utility will increase overall, because her authenticity preference will be better met. Adam’s utility will also improve, because in his new more conservative neigborhood he can also express an attitude more consistent with his own private attitude and will thereby increase his authenticity-related utility. Thus, both Alison and Adam will have an incentive to move to the other’s location, as they will both increase their utility by doing so (see Motyl et al., 2014, for evidence of such behavior). This provides an account of homophily based on utility maximization, quantifying a rational tendency for “birds of a feather to flock together.” We expect it to lead both to segregation and to polarization, when polarization is measured in terms of expressed attitudes. The polarization occurs because agents with extreme views will, if they are initially located in a random position in the network, on average initially be surrounded by more moderate neighbors and hence will moderate their expressed attitudes. After segregation, these agents will express more extreme attitudes of the reasons just described.

We simulate a network of 100 × 100 agents. The simulation involves the following steps. At the start of the simulation each agent is endowed with their own private attitude. This requires specifying both an α and a β parameter for each agent, and proceeds as follows. First, each agent is allocated a mean private attitude, specified to two decimal places, drawn from the distribution beta (10,10), such that moderate attitudes are more common than extreme attitudes. (Using a beta distribution to generate the private attitudes ensures that all lie between 0 and 1.) Beta (10,10) is qualitatively similar to a normal distribution; 63% of the values lie between .4 and .6, and 99% lie between .2 and .8. Each agent is then given the α and β parameters that corresponded to their mean private attitude; this leads to a reasonable distribution of initial attitudes with a variety of precisions. For example, if an agent’s private attitude were .43, it would be assigned α = 43 and β = (100 − 43) = 57. If an agent’s private attitude were .60, it would be assigned α = 3 and β = (5 − 3) = 2. The smallest integers that gave the required private attitude were used. Thus each agent’s private attitude is given by their [α, β] pair; these private attitudes remained constant throughout each simulation reported below except where explicitly mentioned (Demonstrations S2 and S3).

The remaining actions happen on each of many successive cycles of the simulation. In general terms, during each cycle each agent updates their *A*_*i*_ on the basis of the change in expressed attitudes arising on the previous cycle. First, each agent looks at the eight-element vector representing the expressed attitudes of its neighbors. Each agent then fits a beta distribution to that vector, and the estimated parameters of this beta distribution specify the social norm for that agent.^8^ Here and throughout α and β values for inferred social norms are constrained to sum to a maximum of 20; this sets a limit on the precision with which social norms may be represented cognitively and avoids pathological behavior that may otherwise arise if representations of social norms become too narrow and converge toward single points.

Second, each agent calculates the utility-maximizing attitude for it to express, given its (invariant) private attitude and its perception of the social norm. This is done by simulation, using Equation 4 above, and works as described in the previous sections. The expressed attitude of every agent, *A*_*i*_, is then updated to be that agent’s maximum-utility expressed attitude. This updated value becomes the attitude that is observed by the agent’s network neighbors on the next time step.

Third, the mechanism through which agents may move locations is specified. The mechanism was deliberately kept as simple as possible; no intelligent searching of locations by agents is assumed. On each time cycle of the simulation, after a run-in period of two cycles which allows expressed attitudes to stabilize, two agents are chosen at random. Each of the two agents that have been selected looks at the neighborhood of the other agent and calculates the maximum-utility expressed attitude it would express if it were in the alternate location. It then compares the total utility it is experiencing in its current location with the total utility it would experience if it were in the other location. If and only if both of the randomly chosen agents could improve their utility by switching to the other agent’s network location, the two agents exchange places. Otherwise, no switch takes place and another two agents are chosen at random until a utility-improving change is made. The simulation continues to the next cycle when one and only one exchange has been made and when agents’ expressed attitudes have been updated to reflect the switch.^9^

Thus the decision of agents to move to a particular region of the network could be interpreted psychologically as a choice to pay selective attention to (i.e., to expose oneself to) the attitudes expressed in that neighborhood, for example, through a particular set of media. Alternatively, and equivalently from the perspective of our network topography, it could represent a decision to spend more time socially with agents expressing a particular set of viewpoints.

**Figure 11 fig11:**
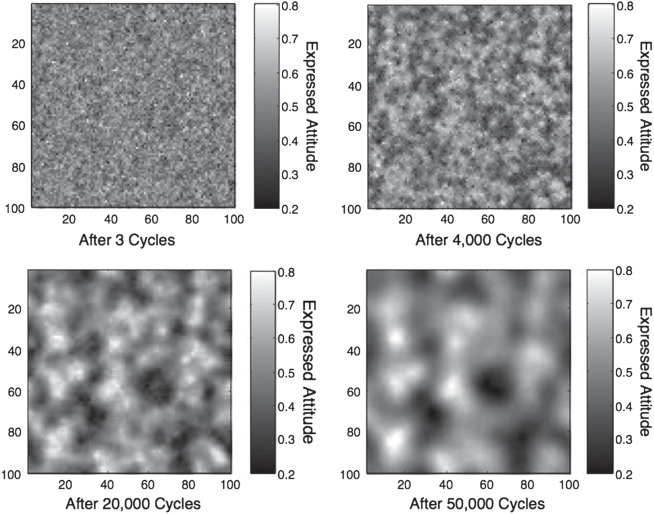
Polarization in the Network Over Time

We focused on two aspects of the network’s behavior: segregation and polarization. We allowed the network, with parameters *w* = .5 and γ = 20 as in the examples discussed above, to run for 50,000 cycles. Figure 11 shows the distribution of attitudes across the network as it evolves over time. The shading of the squares represents the expressed attitude of the agent in the square, with white representing an agent expressing an extreme left-wing attitude (.2) and black representing an agent expressing an extreme right-wing attitude (.8).^10^ The four panels of the figure show the state of the network after 3, 4,000, 20,000, and 50,000 cycles. (Although these numbers may seem unrealistically large, only two of the 10,000 simulated agents change locations on each cycle of the simulation. This implementation is adopted to preserve transparency given complications that ensue when agents make movement decisions based on non-updated environments. In reality, multiple agents would likely change locations simultaneously, and hence the number of cycles should not be taken as representative of real time; even if only 20% of agents change location on each time cycle the network reaches equilibrium in a relatively small number of simulated time steps.)

Over time the network gradually segregates, such that clusters of agents with similar attitudes come to populate particular regions of the network. Agents choose to favor sources of information consistent with their own and hence prefer to surround themselves by individuals whose views are consistent with their own. This enables agents to maximize their utility, as they are thereby able to express attitudes closer to their true attitudes without suffering too much social extremeness. This mechanism offers a quantitative explanation for why rational agents, each with an authenticity preference and an aversion to social extremeness, would tend to flock together.

The fact that segregated clusters can emerge in social networks due to the operation of simple rules for homophily has long been known. The novel aspect of the present approach is the adoption of a cognitively plausible mechanism for rational utility maximization based on a preference for avoiding social extremity, and examination of the resulting polarization to which we now turn.

**Figure 12 fig12:**
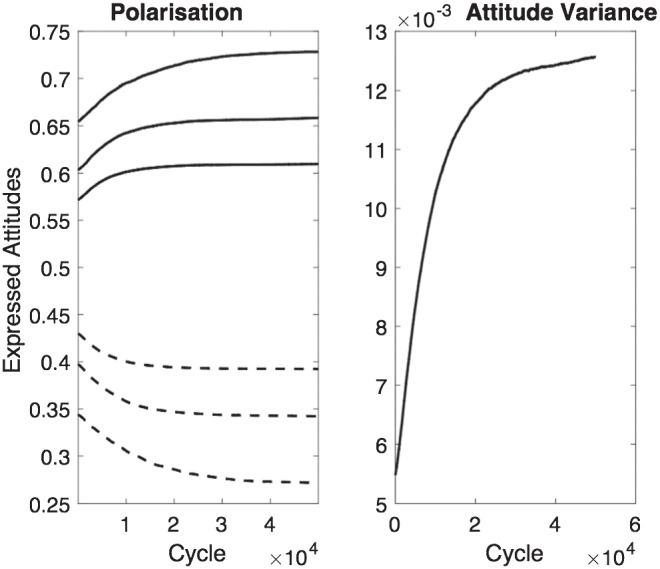
Development of Polarisation in the Network *Note*. Left panel: Evolution over time of the expressed attitudes of various percentiles of the population. Right panel: Attitude variance increasing over time.

Of particular interest are the effects of gradual segregation in the network on the distribution of expressed attitudes across the entire population. The right-hand panel of Figure 12 shows how the variance in attitudes across the network changes over time. Variance increases as segregation occurs, reflecting the fact that agents with relatively extreme private attitudes are able to express more extreme attitudes after they have moved to clusters of others with similar attitudes. The left-hand panel of Figure 12 shows the evolution over time of the average expressed attitudes of the most extreme 5%, 20%, and 40% of the population at either end of the attitude distribution (the lines with the highest and lowest values show the average of the 5% most conservative and 5% most liberal agents, defined in terms of their expressed not underlying attitudes; the lines with values closest to .5 show the equivalent 40% values). At the beginning of the simulation, when no segregation has occurred, expressed attitudes of these agents are drawn toward the middle of the distribution. With increasing segregation, however, agents are increasingly likely to have found a location in the network where they can be surrounded by relatively congenial neighbors and are hence able to express more extreme attitudes. The same is true, to a lesser extent, for agents at less extreme percentiles of the distribution of expressed attitudes.[Fig fig12]

**Figure 13 fig13:**
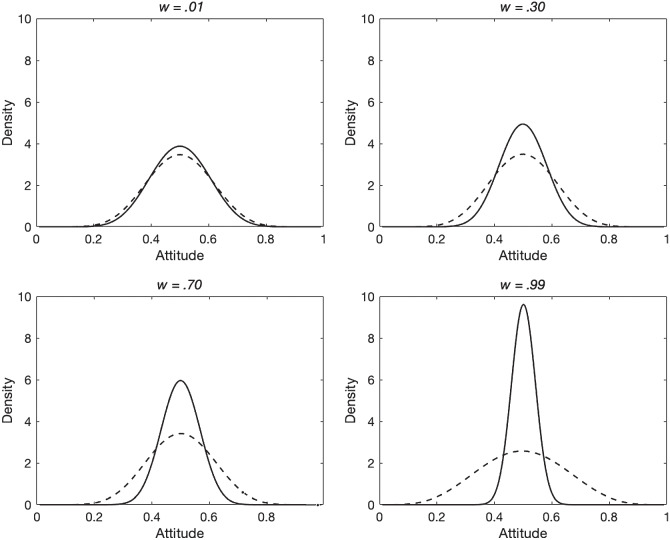
Distribution of Expressed Attitudes After the First Cycle of the Simulation (Solid Lines) and After 50,000 Simulation Cycles (Dashed Lines)

To illustrate the behavior of the model in more detail and explore the robustness of its behavior, we examined the amount of polarization that occurs for different values of the social comparison parameter *w*. It was expected that increased social comparison would lead to the emergence of more polarization, and this is what was found. Figure 13 shows the distribution of mean expressed attitudes at the end of the 50,000 cycles of the simulation for different values of *w* (.01, .30, .70, and .99). For comparison, each panel also shows the distribution of mean expressed attitudes at the end of the first cycle of the simulation. Two key effects are evident. First, after the first cycle of the simulation (i.e., before any agents have had the opportunity to move to more congenial neighborhoods and hence express more extreme attitudes) social comparison leads to a convergence of expressed attitudes. This convergence is greater when the social comparison parameter *w* takes a higher value, and reflects the movement of each agent’s expressed attitude toward the median of that agent’s social neighborhood. Second, by the end of the simulation, more polarization (a flatter distribution of expressed attitudes) is seen when social comparison is greater. This is because movement into neighborhoods where the social norm aligns with a private attitude has more effect when social comparison is high (if there were no social comparison, i.e., *w* = 0, there would be no effect of social neighborhood on expressed attitudes whether or not agents changed their locations).[Fig fig13]

As a robustness check, we examined the extent of polarization under various different parameter combinations. Specifically, we varied γ (values between 5 and 50), *w* (values between .01 and .99) and the α and β parameters that characterize the initial distributions of attitudes (values between 3 and 30). For each combination of parameters, we calculated total disutility (summed across all agents; see Demonstration 2.2 below) at the end of the simulation, and the degree of polarization that emerged (measured as the ratio of the variance in mean attitudes at the end of polarization to the variance in mean attitudes before any polarization occurs). Twenty replications of the simulation were conducted for each set of parameter values to examine the consistency of results.

The results are shown in Table 2. It is evident from the low standard deviations that similar results were obtained over the 20 replications for each parameter combination. Polarization was observed under all parameter value combinations, but did not vary greatly for different parameter values with the exception of the large effect of *w*; higher values of *w* lead to greater polarization (as also shown in Figure 13 and discussed in that context).[Table tbl2]

We also examined robustness of the polarization to changes in the functional form relating disutility to extremeness (Equation 3). The results are described in S4 (in Supplementary Online Material), and were very similar to those obtained with the standard (convex) function.

#### Demonstration 2.2: Aggregate Network Well-Being

**Figure 14 fig14:**
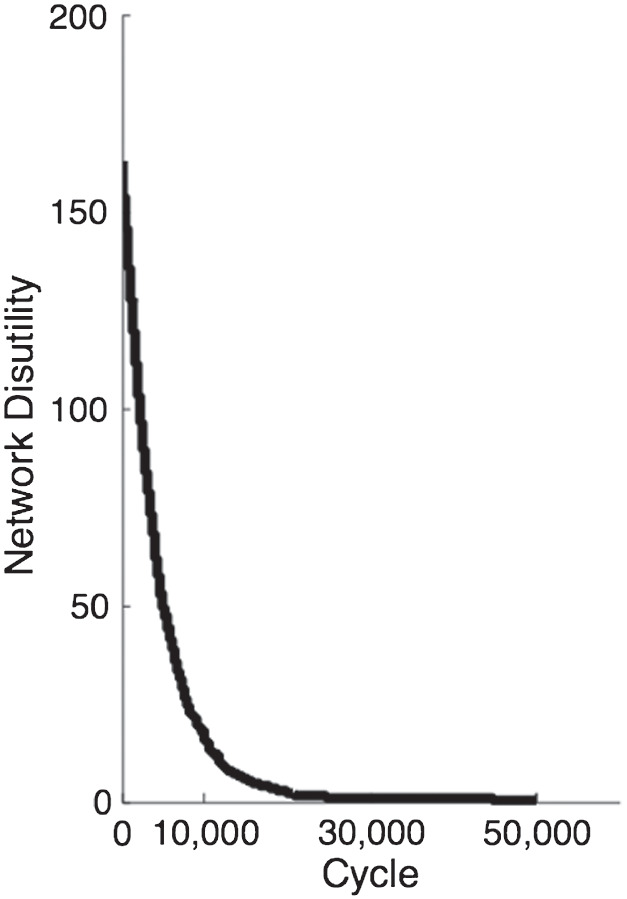
Network Disutility Reducing (Welfare Increasing) Over Time

A consequence of polarization is that the average well-being in the network will increase gradually over time as segregation and polarization occur (i.e., disutility will reduce). This is because agents lose less authenticity-related utility as they move and become less constrained (by social extremeness aversion) to express a less authentic attitude. The effect is illustrated in Figure 14, which shows the reduction in total network disutility over time as polarization occurs (the same parameters as first simulation above).^11^ As shown in Table 2, the disutility reduction is robust across a range of parameter values, although the absolute amount inevitably depends on parameter selection.[Fig fig14]

#### Demonstration 2.3: Social Contagion Effects

Next, we examine the conditions under which attitudes expressed by a single agent can spread through networks. There is a large literature, spread across many disciplines, on the effects that zealots or committed minorities can have on wider opinion (e.g., Masuda, 2012; Moscovici, 1980; Verma et al., 2014); our aim here is to show how and when mechanisms may give rise to such effects.

In this simulation, the network structure remains the same as in previous simulations, as do the principles governing the behavior of each agent within the network. We use a smaller (20 × 20) network, because each simulation takes a long time to run and the spread of attitudes is no better explained by larger networks. As before, each agent observes the expressed attitudes of its immediate neighbors on each time cycle, and on the basis of those attitudes and its own randomly selected fixed private attitude the agent expresses its utility-maximizing attitude. However, unlike in previous simulations, agents never change position and hence the network stabilises after only a few cycles. On the tenth cycle, however, we place a single new agent into a random location in the network. This agent (the “seeded agent”) differs from other agents in two ways. First, it is endowed with an extreme attitude (.99). Second, it is a “stubborn agent” (Acemoglu & Ozdaglar, 2011) and is immune to social norms; it expresses the same attitude of .99 on every cycle of the network no matter what attitudes are being expressed by its neighbors.

The question of interest is whether—and under what conditions—the expressed attitude of the seeded agent’s neighbors will spread throughout the network, influencing first its immediate neighbors, then the neighbors of those neighbors, and so on. To illustrate as simply as possible, we set *w* = .8 for all agents (except the agent that is seeded into the network, for which *w* = 0), and varied γ. Initial endowment of private attitudes was as in previous simulations.

**Figure 15:**
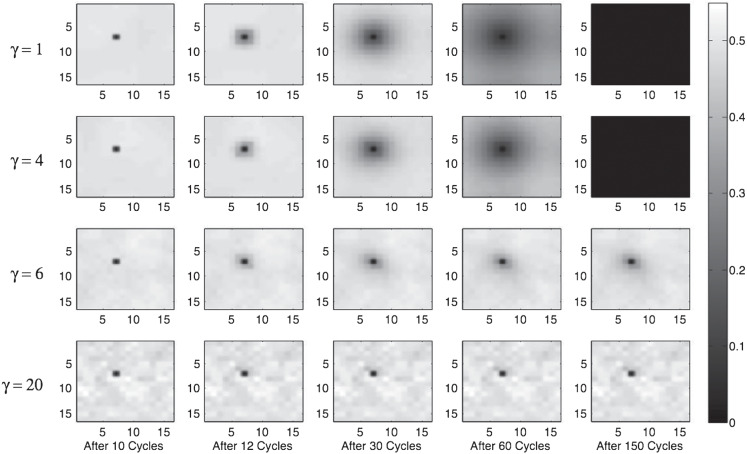
Social Contagion Effects Over Time as a Function of Parameter w

Results are illustrated in Figure 15. Three qualitatively different patterns of behavior are seen, corresponding to different values of γ. When γ is small (less than ≅ .5; top two rows of panels), strong social contagion occurs. The expression of the extreme attitude propagates through the network, spreading outward from the seeded agent until eventually every agent in the entire network is expressing the same, extreme, attitude. When γ is around 6, however, social contagion occurs but is limited in extent: Only agents in the immediate social neighborhood of the seeded agent are influenced in the direction of expressing a more extreme attitude, and the effect is diminished as the social distance from the seeded agent increases. This pattern is illustrated in the third row of panels in Figure 15. Finally, as γ becomes larger (e.g., 20), no social contagion occurs.^12^

Why does γ influence social contagion? Recall that γ governs the rate at which disutility increases as authenticity is lost and social extremeness increases (Figure 4A and Equation 3). The higher the value of γ, the more extreme an expressed attitude must be to give rise to significant disutility. For example, when γ = 4, 50% of the maximum possible disutility related to social extremeness occurs when an expressed attitude is at about the 83rd percentile of the social norm, and 80% of the maximum possible disutility occurs when an expressed attitude is at about the 94th percentile of the social norm. When γ = 20, in contrast, expressed attitudes must be at about the 96th and 99th percentiles of the social norm to attract the same amounts of disutility. In other words, the greater the value of γ, the more tolerant agents are of a given level of social extremism. Specifically, the effect of the seeded agent’s extreme opinion on its neighbors’ *estimates* of how socially extreme they are will be independent of γ. However, if γ is low, the *effect* of those estimates on the attitudes that are utility-maximizing for an agent to express is greater. The greater the effect on immediate neighbors, the greater the effect on the neighbors of those neighbors, and so on. It is useful to consider the limiting case: When γ reduces to zero, agents will incur the maximum extremeness-related disutility whenever they express an attitude that is anything except the exact median of the social norm (see Equation 3).

#### Demonstration 2.4: False Consensus Effects

An important factor relating to social norms and polarization is the perception of one’s immediate social environment (Galesic et al., 2012, 2013, 2018; Pachur et al., 2013; Schulze et al., 2020). People typically overestimate the prevalence of their own opinion in a population—this is the *false consensus effect* (e.g., Leviston et al., 2013; Marks & Miller, 1987; Ross et al., 1977), with the effect being stronger for people who hold minority opinions (Krueger & Clement, 1997).

**Figure 16 fig16:**
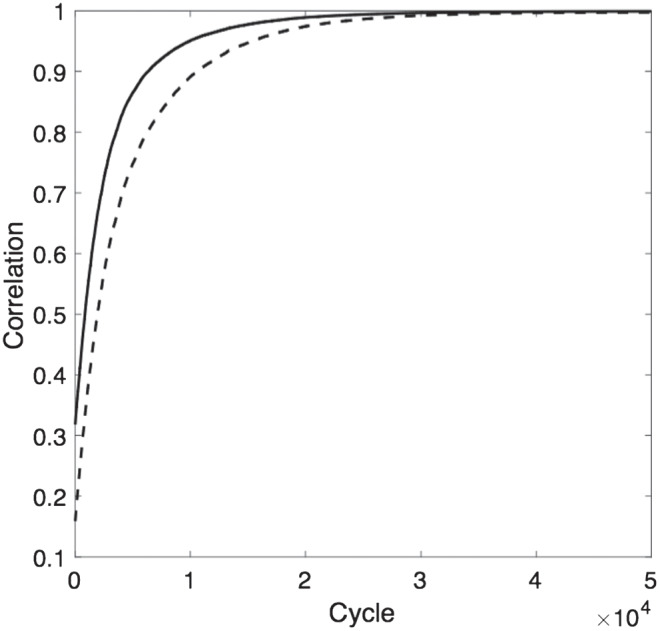
Emerging False Consensus Effects *Note*. False consensus effects over time, represented as the correlation between the mean expressed attitudes of each agent’s neighbors and that agent’s expressed (solid line) and private (broken line) attitudes

We examined the emergence of false consensus as polarization occurs. We assume that agents estimate the opinion of other agents by taking the mean of the social norm that they have estimated on the basis of the attitudes expressed by their eight immediate network neighbors. As segregation occurs, these neighborhood means will tend to converge toward the opinions of the estimating agent. Figure 16 plots the simple correlation between the mean expressed attitudes of each agent’s neighbors and that agent’s expressed (solid line) and private (broken line) attitudes. As expected, the correlations increase as the simulation progresses and segregation occurs. This occurs because each agent gradually locates itself within a neighborhood of similar others, hence (by design) increasing the similarity between its own attitudes and the expressed attitudes of immediate neighbors.[Fig fig16]

Thus false consensus effects fall naturally out of SST (although we note the existence of several alternative explanations: Galesic et al., 2013, 2018; Marks & Miller, 1987). In SST, false consensus effects will arise whenever segregation occurs and people over-sample from local regions of their social networks when estimating population attitudes. For robustness, we examined the effects of parameter variation on the emergence of false consensus effects. As shown in Table 2, false consensus effects occur under all combinations of parameter values that we examined.

#### Demonstration 2.5: Pluralistic Ignorance Effects

A counterpart to the false consensus effect is *pluralistic ignorance*—pluralistic ignorance is typically said to be present when individuals holding the majority opinion incorrectly believe themselves to be in a minority (e.g., Prentice & Miller, 1993; Shamir & Shamir, 1997). For example, Todorov and Mandisodza (2004) found that American citizens mostly preferred multilateral foreign policies but that many of them incorrectly believed that a majority of others supported unilateralist approaches. A related phenomenon is the false uniqueness effect, which occurs when people believe that their own view is less widespread than it actually is (Frable, 1993).

In SST, pluralistic ignorance occurs under similar conditions to those that produce strong social contagion. We illustrate by endowing agents with an asymmetric distribution of private attitudes. Specifically, we create a population whose mean private attitudes are drawn from the distribution beta (8,10) instead of the symmetrical distribution beta (10,10) used in earlier simulations. As a result, about 70% of agents have private opinions with *M* < .5, and “*M* > .5” is therefore the minority opinion. We assume, as we did when illustrating the false consensus effect, that agents estimate the opinions of the entire populations by sampling from their local neighborhood. Specifically, on each time cycle of the simulation each agent counts up the proportion of their eight network neighbors who are expressing a view >.5, and uses that proportion as an estimate of the population proportion.

**Figure 17 fig17:**
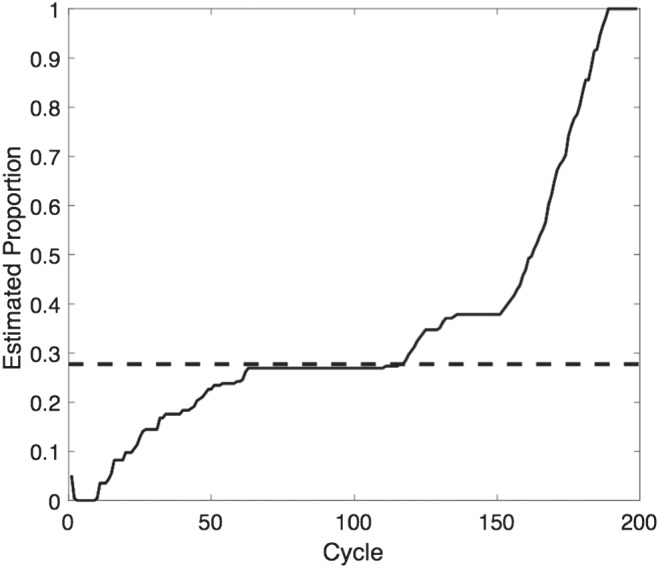
Pluralistic Ignorance Effects Over Time

As with our contagion simulations, on the tenth time cycle of the simulation we introduce one agent with a fixed expressed view (.99), which in this case corresponds to the minority opinion. As this agent’s extreme opinion gradually spreads through the social network, an increasing number of the other agents become surrounded by neighbors expressing an opinion >.5 and their estimate of the proportion of the population holding such an opinion increases. The process is illustrated in Figure 17, which was obtained with γ = 1 and *w* = .9. The vertical axis shows the average of agents’ estimates, based on the attitudes expressed by their local neighbors, of the proportion of the population holding an opinion >.5. The horizontal line represents the (unchanging) proportion of agents in the network whose mean private attitudes are actually >.5.[Fig fig17]

In the initial few cycles of the simulation, the expressed views of most agents move toward the majority (<.5) opinion. On the tenth cycle, the agent with an extreme view is introduced, and consequently that agent’s neighbors and then the neighbors of those neighbors gradually come to express views >.5 themselves even if their private views are <.5. As the proportion of agents expressing >.5 increases, the average estimate of that proportion increases and with parameters that lead to complete social contagion (see Figure 15) eventually reaches 1.0. Thus all the agents privately holding what is in fact the majority view (i.e., <.5) eventually come to believe that all other agents hold the opposite view (i.e., >.5), because that opposite view is what neighboring agents have come (due to social pressure) to express (cf. Kuran, 1995). The simulation therefore illustrates one way in which pluralistic ignorance may occur in SST.

In additional simulations (reported as S3 in Supplementary Online Material), we extend this approach by investigating the effects of exposing every agent, on every time cycle, to a consistent set of opinions expressed by other agents. These consistent opinions can be thought of as those represented in media sources or by political leaders (Bail et al., 2018). The simulations show, consistent with intuition, that both private and expressed attitudes converge toward those expressed by the consistent agents whose attitudes are seen by everybody.

## General Discussion

SST joins a large set of models that have sought to explain various aspects of polarization, social influence, and opinion formation in social networks (for reviews, see, e.g., Acemoglu & Ozdaglar, 2011; Lehmann & Ahn, 2018; MacCoun, 2017). We have already noted social sampling models (e.g., Galesic et al., 2012, 2018; Pachur et al., 2013) and ABMs focussing specifically on polarization (e.g., Flache & Macy, 2011b; Latané & Wolf, 1981; Nowak et al., 1990). There are many other models of polarization from various disciplines (e.g., Andreoni & Mylovanov, 2012; Baumann et al., 2020, 2021; Cook & Lewandowsky, 2016; Jern et al., 2014) as well as models of how media coverage may be influenced by polarization (Bernhardt et al., 2008). Numerous relevant models of social influence and opinion spread have also been developed in other disciplines, such as economics (Akerlof, 1980; Bernheim, 1994; Chamley, 2003; Jones, 1984), sociology (Friedkin & Johnsen, 2011), ecology (Bentley, Ormerod, et al., 2011), marketing (Iyengar et al., 2011; Rao & Steckel, 1991), and the study of complex systems (Deffuant et al., 2000; House, 2011; Sznajd-Weron & Sznajd, 2000). We have not been able to do justice to these or to a large literature on the psychological principles underlying the spread of mass opinion (e.g., Zaller, 1992). Given that space limitations preclude a comprehensive review, here we first delineate the features of SST that distinguish it from other models and summarize how those features give rise to its behavior, then discuss predictions, limitations and possible extensions, and implications.

### Key Distinctive Features of Social Sampling Theory

#### Social Extremeness Aversion and Authenticity Preference

Most of the properties of SST emerge as a result of the interaction between two opposing factors: social extremeness aversion and authenticity preference. Any model of polarization must explain both (a) the continued existence of individual differences—networks do not always converge to homogeneity—and (b) the tendency of expressed attitudes to become more extreme when agents are able to choose their social or informational neighborhoods (Abelson & Bernstein, 1963). Although these are distinct phenomena, in SST authenticity preferences (together with individual differences in private attitudes) underlie both. It is the authenticity preference that prevents the network from converging to homogeneity and allows differences in attitudes to persist in the face of social influence, while SST’s social extremeness aversion leads to homophily and polarization. In this respect SST differs from models that ascribe polarization to a preference for, or selective influence by, extremeness (Abelson & Bernstein, 1963).

We have remained neutral on the underlying reasons for the existence of social extremeness aversion; there are many (nonexclusive) plausible distal causes of conformity (e.g., Kelman, 1958). However, we note particularly that, in addition to supporting coordination, social conformity may be adaptive when others have information that the conforming agent does not (Bentley, Earls, et al., 2011; Chamley, 2003) rather than simply reflecting a taste for conformity per se (Deutsch & Gerard, 1955). In one interpretation of SST, therefore, (a) people have uncertainty about their private attitudes, which can be seen as a type of preferences, (b) they assume that they are similar to other people, as false consensus effects suggest, and (c) they assume that the “market” of expressed attitudes in their social environment reflects the aggregate private attitudes of the population. Effects of social norms can then be interpreted as part of the process of inferring one’s own attitudes from a combination of an uncertain private signal and the expressed attitudes of (assumed similar) others who are assumed to have additional sources of information.

We note a possible relationship between the ideas presented here and the concept of cognitive dissonance (Festinger, 1954). According to cognitive dissonance theory, inconsistency between actions and attitudes is negative for well-being, and people are motivated to reduce the discrepancy between them. If we interpret the expression of attitudes as the “actions” of cognitive dissonance theory, and the “attitudes” of cognitive dissonance theory map onto the private rather than the expressed attitudes of SST, our proposal can be seen as a simple implementation of cognitive dissonance. However, we differ from, for example, Festinger and Carlsmith (1959) in emphasizing the role of social norms, rather than compliance induced by other means, in leading to expressed attitudes that differ from those that are more “authentic,” and we also note that dissonance theories typically (but unlike SST) assume conscious awareness of discrepancies between attitudes and behaviors.

We also note that the processes specified in SST may underpin one form of “deliberate ignorance” (Golman et al., 2017; Hertwig & Engel, 2016) in that people’s authenticity preferences may motivate their avoidance of individuals (or other sources of information) who espouse, or otherwise represent, attitudes incongruent with one’s own. Authenticity preference can also be seen as underpinning preferences for belief consonance (Golman et al., 2016) and maintenance of a consistent identity (Bénabou & Tirole, 2011; Golman et al., 2017).

#### Attitudes Are Distributions

Attitudes are represented as distributions, not single points, in SST to reflect the fact that any given attitude may be more or less precise. The attitudes-as-distributions assumption is central to SST as it underlies its ability to account for the effect of attitude precision on social norm effects and backfire effects; a person whose attitude precision is high will be less influenced by social norms, and is more likely to show backfire effects, because the cost they suffer (in terms of authenticity loss) of conforming is higher for a given amount of conformity. In its emphasis on distributional representations of attitudes SST therefore goes beyond models which represent opinions as binary (e.g., Nowak et al., 1990) or as single points on a continuum (e.g., Dittmer, 2001). The key claim is that the distributional properties of attitudes must be represented in some way; we make no claim that our specific implementation (using beta distributions) is the only way this could be done.

The claim that attitudes are distributions is not the same as the claim that attitudes are noisy or uncertain; people may occupy the same ideological position but differ in how committed they are to that position—informally, they may differ simply in how strong their preference for a given position is. The claim that attitudes are distributions is also different from the idea that there are differences in attitude-related “awareness” as defined by Zaller (1992), in the context of political attitudes, in terms of the amount of attention to and understanding of relevant issues. The claim is also distinct from the notion of an attitude’s “importance” to satisfaction or value achievement (Rosenberg, 1956) and in addition differs from the idea that binary attitudes might differ in the strength with which they are held (e.g., Bassili, 1996).

#### Social Norms Are Distributions

SST assumes that social norms, like attitudes, are best viewed as distributions. The estimation of social norms, as it occurs in SST, can be seen as a normative estimation of public attitudes given a sample. This assumption that the social norm is a distribution distinguishes the approach from a number of models in economics, health psychology, and consumer science, which typically assume that norms can be represented as single points such as a typical wage, reference, price, weight, or level of alcohol consumption. As with the assumption that attitudes are distributions, the treatment of social norms as distributions rather than single points is central to the behavior of SST. This is because the assumption allows for effects of consensus in the social norm—a person will need to conform more in the direction of a social norm to achieve a given reduction of social extremeness aversion when there is high consensus in the social norm (i.e., when the estimated social norm has high precision). Moreover, backfire effects can emerge when social consensus is high. Neither of these effects would emerge in SST without the assumption that social norms are distributions, not single points.

#### Concern With Relative Rank

Another key distinguishing feature of SST—closely related to its distributional assumptions—is that people care about where they rank within a social distribution rather than how they relate to the mean of that distribution. As noted earlier, a large body of empirical work on people’s judgments of quantities, such as their exercise levels, alcohol consumption, and so on, supports this assumption, and the claim is also consistent with rank-based sampling models of judgment and decision-making (Bhui & Gershman, 2018; Stewart et al., 2006).

#### Distinction Between Private Attitudes and Expressed Attitudes

The distinction between private attitudes (which in most demonstrations above are assumed to be fixed and unchanging characteristics of an individual) on the one hand, and expressed attitudes (which can change as a function of social context) on the other, underpins much of SST’s behavior and can be seen as both a strength and a weakness. The existence of fixed individual characteristics is consistent with the idea that some individual differences in values (e.g., ideology) are relatively stable characteristics of a person over their lifetime. The assumption offers one explanation for why social norm effects do not cause people’s attitudes to converge over time and eventually become identical; the fixed private attitudes provide an essential “opposing force” which counteracts effects of social norms. SST’s explanation of why areas of non-convergent opinions may survive contrasts with others that have been given in terms of network structure and noise (e.g., DiFonzo et al., 2013; Mäs et al., 2010). We also note that our conception of private attitudes resonates with the concept of “attitude roots” developed by Hornsey and Fielding (2017), and that the assumption of both (assumed stable) private attitudes and (socially influenced and context-dependent) public expressions of attitudes relates to a long-standing debate about the stability of political attitudes (Converse, 1964; Diener, 1975; Zaller, 1992). A distinction between private and expressed preferences can also be found in voting models (Gastner et al., 2018; Masuda et al., 2010).

We do not assume that agents have direct and privileged access to their own private attitudes. Instead, we regard SST as one possible implementation of the idea that people infer their attitudes from their own overt behavior (Bem, 1967; Wilson, 2002; cf. also Nisbett & Wilson, 1977). However, the operation of SST as we have presented here does not hinge on whether or not private attitudes are accessible to conscious awareness. There is in any case a need to distinguish between, on the one hand, cases where one explicitly conforms in the sense of expressing or assenting to views that one knows one does not truly hold, as in “preference falsification” (Kuran, 1995) and, on the other hand, cases where one genuinely believes oneself to be holding the view that one expresses notwithstanding the fact that one might have believed oneself to hold a different view if circumstances were otherwise. Thus a complete model will likely need to distinguish between (a) *private*/*authentic/underlying* attitudes, which are only indirectly accessible to awareness, (b) *inferred* attitudes, which are what people believe their private/authentic/underlying attitudes to be, and (c) *expressed* attitudes, which are reflected in overt behavior and are observable by others. The mechanisms describe in the present paper could therefore be seen as reflecting the informational role of social norms (whereby an individual’s beliefs about their own attitudes are informed by observation of others’ attitudes), and/or as an account of the social conformity that causes a person’s expressed attitudes to differ from their attitudinal beliefs.

#### Double-Peaked Preferences

Unlike most models within economics and political science, SST allows for the possibility of double-peaked preferences. This can occur when the utilities associated with expressing either one’s authentic attitude, or conforming to the social norm, both exceed the utility associated with expressing a compromise attitude. To motivate the intuition (albeit with an example concerned with beliefs rather than attitudes) consider participating in a social conformity experiment (Asch, 1956) in which one must report whether the length of a line is 1 or 3 m. One’s own perceptions indicate strongly that the line is 1-m long, but the other participants in the experiment all report that it is 3-m long. In such a situation, there is little to be gained by compromising and suggesting that the line is 2-m long; rather, locally utility maxima correspond to being true to one’s own beliefs (and reporting 1 m) or conforming completely (reporting 3 m). The possibility of double-peaked preferences is responsible for some of the sudden changes in expressed attitude in SST; these occur when one peak suddenly becomes higher than the other leading to a sudden switch in expressed behavior as a function of a smoothly varying parameter (see Demonstration 1.3).

### Predictions

Our aim has been to (a) account for a range of existing phenomena with as simple a model as possible, while (b) using basic building blocks that are independently motivated by evidence from relevant areas of psychology. But is SST falsifiable? In this section, we summarize novel predictions from the model, along with suggestions for how they might be tested in future work. Predictions fall into two categories. On the one hand, as we have illustrated throughout, SST makes predictions about how particular effects will vary as a function of parameter values (see, e.g., Table 2). An important issue is therefore the feasibility of measuring individual differences in those parameter values, and we address that issue here. Other SST-specific predictions result from the aspects of the model architecture that distinguish it from most previous models as outlined in the previous section, and we therefore organize this section in similar fashion.

Predictions that most clearly distinguish SST from other models arise from its assumptions that both attitudes and social norms are represented as distributions rather than single points, and that the width of these distributions matters for behavior. At the most general level, these assumptions lead to the idea that the feelings of authenticity or social extremeness associated with expression of a particular attitude will depend not on the distance of the expressed attitude from a social norm or authentic attitude that is represented by a single point, but rather by the position of the expressed attitude within the relevant distribution. Thus SST predicts that narrower (more precise) representations of attitudes and social norms will lead to the expression of attitudes that are closer to the medians of the distributions representing private attitudes and social norms respectively, but are also predicted to be more susceptible to backfire effects (see Demonstration 1.3). Can the locations and precisions of private attitudes and perceptions of social norms be measured? In the case of perceptions of social norms, relevant methodology already exists. In a number of previous studies, we have shown how people’s beliefs about social norm distributions (of, e.g., exercise levels or the consumption of alcohol or unhealthy food) can be elicited by asking people to estimate percentile points of the relevant social distributions (Aldrovandi, Brown, et al., 2015; Maltby et al., 2012; Wood et al., 2012). Given this knowledge of an individual’s beliefs about the social norm, it should be possible to predict how a person’s expressed attitude will change as their beliefs about the social norm (which may be incorrect due to sampling bias), and hence the position they believe their expressed attitudes to occupy within that distribution, are changed. Related experiments have already been reported in other domains. For example, telling people their true relative ranked position in the social norm of unhealthy food consumption increases the premium they are prepared to pay for a healthier food option by an amount that depends on the initial degree of people’s misperception of their relative ranked position within the social norm (Aldrovandi, Brown, et al., 2015), and telling people where their level of alcohol consumption ranks within the social norm increases relevant information searching more than does telling them how their consumption relates to the mean of the social norm (Taylor et al., 2015). Similar methodologies may be applied to test SST’s predictions regarding the expression of attitudes more generally.

The measurement of private attitudes is more difficult, given SST’s assumption that overt behavior reflects expressed rather than private attitudes. There is already a large literature on the measurement of implicit attitudes, and on the ability of such measures to predict behavior over and above measures of explicit attitudes (see, e.g., Schimmack, 2019, for a review). Psychophysiological measures might also hold potential, as might reaction time measures (Bassili, 1996). However, there are important differences between the private attitudes of SST and implicit and/or unconscious attitudes as typically conceived of. Moreover, it is difficult to see how the precision of implicit attitudes can be assessed. In the light of these considerations, we view the distributions that represent private attitudes as theoretical quantities that can be inferred but not measured directly.

Another set of predictions concerns the relative importance of authenticity preference and social extremeness aversion. SST predicts that agents with stronger authenticity preference and/or weaker social extremeness aversion should be more susceptible to polarization. Measures of different components of authenticity already exist (Wood et al., 2008), with one component being “accepting external influence,” although measures of authenticity are of course conceptually distinct from measures of authenticity preference (one could be inauthentic but wish to be authentic). However, it seems reasonable to assume that people are authentic because it is important to them to be so, in which case SST would predict that high-authenticity individuals will be less susceptible to social influence in general and polarization in particular.

Further predictions arise from the claims that SST makes regarding well-being. Granted the assumption that subjective well-being (i.e., self-reported life satisfaction or positive and negative affect) will in part reflect discrepancies between expressed attitudes and both private attitudes and the social norm, it should be possible to predict subjective well-being from the discrepancy between expressed attitudes and the perceived social norm (which can be elicited as described above). Specifically, SST predicts a negative association between subjective well-being and the distance (in rank space) between an attitude that an individual expresses and the median of the social norm that the individual believes to obtain.

We also highlight distinctive predictions arising from the possibility of double-peaked functions describing the utility associated with expressing particular attitudes. In SST, double-peaked functions occur when it is worse for an agent to express a compromise attitude than to express attitudes closer to the medians of the distributions representing their authentic attitudes and the social norm. Double peaks (and backfire effects) are more likely to occur in SST to the extent that (a) there is a large difference between the medians of the two distributions, and (b) the distributions are precise. It should in principle be possible to test this prediction by eliciting the attitudes people prefer to express while varying a hypothetical social norm.

A final class of predictions concerns ease of transmission of attitude-congruent and attitude-incongruent messages through social networks. There is already a large body of research on how the transmission of both information and misinformation through social networks depends on the features of the message such as its novelty (e.g., Vosoughi et al., 2018). One key prediction of SST is that (other things, such as network structure, being equal) messages that are congruent with private attitudes will be transmitted from one agent to the next, and onward throughout the network, with less distortion than attitude-incongruent messages. This is because agents will under most circumstances transmit to neighboring agents a message (i.e., an expressed attitude) that is closer to their private attitude than was the message they received.

### Limitations and Extensions

#### Network Structure

The simple network architecture that we have adopted, in which all agents see and are influenced by all and only their eight immediate neighbors, is clearly unrealistic as a model of actual social structure. In reality, a few people have a large number of social network connections while most have few (Fowler et al., 2009), and “small world” networks also contain a proportion of long-distance connections (Watts & Strogatz, 1998). Thus, the assumption that all agents are connected to the same number of other agents represents a considerable simplification of the structure of real social networks (Albert & Barabási, 2002; Barabási, 2009). We also acknowledge the fact that features such as long-range connections may influence network behavior in ways relevant to social phenomena such as polarization (e.g., Flache & Macy, 2011a). We believe that the simple network structure we have used enables the best and least obscured illustration of the operation and consequences of the psychological processes embodied in the model. However, it is important to acknowledge that network structure can make a difference to a number of network-level social phenomena (such as the spread of innovations: Abrahamson & Rosenkopf, 1997). Opinions, like behavior, spread differently in different network structures (e.g., Centola, 2010), and structure also influences the formation of echo chambers (e.g., Madsen et al., 2018) and the transmission of extreme opinions (e.g., Amblard & Deffuant, 2004; Franks et al., 2008). The size of the local neighborhood is also important (e.g., MacCoun, 2012). SST does not distinguish between “strong ties” and “weak ties” (Granovetter, 1973), and this distinction may be relevant to understanding polarization (Flache & Macy, 2011b). An important task for further work is therefore to integrate the core mechanisms of SST into models of network *formation*. In initial work along these lines, we^13^ have started to examine the consequences for the development of network structure of assumptions concerning the formation and deletion of edges between individual agents as a function of the discrepancy between private attitudes and expressed attitudes. As intuition (and much previous research) suggests, the incorporation of such assumptions into models of network formation leads to the creation of subgroups of like-minded agents and to the emergence of polarization. However, the mechanisms that underlie the core phenomenon of polarization, as we have defined it in the present paper, remain largely unchanged.

A further, and related, avenue for future research concerns the role of node centrality in opinion contagion; this is an area where SST may make more distinctive predictions as noted above. Specifically, if a node is central to a network in that many of the shortest pathways between pairs of other agents pass through it, then the private attitude of that agent will likely have a larger effect on the expressed attitudes of other agents and will, on average, exert a dampening effect on the extremity of expressed attitudes. Node in-degree is also important; agents who get input from many other agents may have more social influence (Battiston & Stanca, 2015).

#### Influence of Group Identity

Group identity is undoubtedly important in attitude formation and change (Clarkson et al., 2013). However, SST does not represent group structure beyond the segregated clusters that emerge and produce polarization and hence does not allow for greater influence from (otherwise-determined) group members, let alone the interplay between intergroup distinctiveness and differentiation (Jetten et al., 2001) or the different responses of peripheral and central group members (Jetten et al., 1997a, 1997b). We view this as both a limitation and an avenue for future research, and in the latter context note the existence of models that allow for effects of group structure on social contagion (Iacopini et al., 2019).

#### Differences in Attitudes and Extent of Polarization

SST models agents with only one attitude, and this is an important simplification because the multiple attitudes that real agents possess differ in the extent of polarization they are associated with. Indeed, we have assumed a simple unidimensional continuum to be the only dimension on which private attitudes and social norms differ. Moreover, the extent which polarization exists is often overestimated (Baldassarri & Bearman, 2007; Barberá et al., 2015; Fiorina & Abrams, 2008; Westfall et al., 2015), as is the role of social media in polarization (Boxell et al., 2017; Gentzkow & Shapiro, 2011) and the idea that people pay selective attention to viewpoint-consistent media (Dubois & Blank, 2018). Attitudes appear more likely to polarize when they involve “take-off issues” (Baldassarri & Bearman, 2007), typically those relating more closely to fundamental ideology and values rather than, for example, sports and entertainment (Barberá et al., 2015). There may even be relevant differences between people who occupy different locations along a single attitudinal continuum. For example, liberals and conservatives seem to differ in the extent to which they see their values as being similar to those of other people within their political in-group (Boutyline & Willer, 2017; Stern et al., 2014).

How might such effects be incorporated in SST? Our simple implementation inevitably cannot do justice to the full richness of different psychological conceptions of what attitudes really are, and there are many such views (Albarracín et al., 2005). Set against this, an advantage of taking a computational approach as we have done here is that one is forced to be precise about what one means by an “attitude” because it must be specified in the implementation. Within the framework described, it is straightforward to represent differences in (a) the extent of social comparison associated with a given attitude and (b) the precision of different attitudes. Thus, if agents are assumed to possess multiple attitudes, it is plausible that *w* is higher for some attitudes than others (depending, e.g., on how central the attitudes are to either group or individual identity). It is also plausible that attitudes will vary in their precision, and both precision and *w* will influence the importance of attitudes for behavior (Howe & Krosnick, 2017).

#### Social Norm Inference

We have assumed that agents infer social norms in their network neighborhood by fitting a distribution to the attitudes expressed by their immediate network neighbors (or, at least, behaving “as if” they are doing so), and we have assumed this process to be unbiased. This assumption is undoubtedly a simplification in a number of ways. Much research has examined the processes that underpin people’s sample-based inferences about, and knowledge of, distributions. One question is whether such estimates are accurate, and examinations of this question have produced mixed results (Griffiths & Tenenbaum, 2006; Nisbett & Kunda, 1985), with, for example, findings of a bias toward unimodal estimates under some conditions (Lindskog et al., 2013b). Another question is whether people’s estimations of distributions take the form of continually updated estimates of the distribution’s parameters, or are based on small samples retrieved at the time the distribution must be estimated. Most evidence supports the latter assumption (Lindskog, 2015; Lindskog & Winman, 2014; Lindskog, Winman, & Juslin, 2013a), consistent with the idea that people are naïve intuitive samplers (Juslin et al., 2007). Here, we make no strong commitment about *when* social norms are estimated; all that is essential for SST is that people have access to knowledge about the prevailing social norm at the time they decide what attitude to express, and that the knowledge they have is sufficient to enable estimation of their relative rank. SST assumes that the expressed attitudes of all eight social network neighbors inform people’s estimation of the neighborhood social norm; people infer at least something about distributions from as few as four data points (Lindskog, 2015), and the assumption that eight observations are considered does not seem too inconsistent with small-sampling assumptions.

We have assumed that people have complete and full access to (or memory of) the expressed views of people surrounding them; we therefore ignore the possible effects of memory limitations (which may be important in, e.g., explaining false consensus and false uniqueness effects: Galesic et al., 2013) and the search strategies that underpin social recall (Hills & Pachur, 2012). We have not included any “smoothing” of the characteristics of observed others, as Galesic et al. (2012) do in their social sampling model, and, more generally, we have not specified the nature of social sampling in detail (cf. Pachur et al., 2013; Schulze et al., 2020).

A related issue concerns the properties of the scale along which the social norm is represented. Unlike the private attitude distributions, which SST generally takes to be fixed and to have ratio-scale properties, the social norm estimates are based on observations (of the attitudes that other people express). We have assumed that such observations are unbiassed. However, the subjective judgment of any one observation, taken in isolation, is likely to be affected by the context of other observations as would be predicted by most cognitive models of judgment. If the social norm is estimated from these contextually influenced judgments of neighbors’ attitudes, the estimate will be flattened or distorted relative to the estimate that would be based on undistorted perceptions. We have not explored the consequences of such context effects in the present simulations, in part because they seem unlikely to affect the model’s behavior in any systematic qualitative way under the circumstances we have considered, and in part because we believe (although have not justified here) that contextualized subjective judgments are based on precisely the type of distribution-estimation that we have already assumed.

#### Other Psychological Mechanisms

We have aimed to keep SST as simple as possible, and SST has just two free parameters (*w* and γ) beyond the parameters specifying the beta distributions that characterize both private attitudes and the social norm. An additional learning rate parameter is used for explorations of convergence between private and expressed attitudes. We have opted to keep the model as simple as possible because additional mechanisms are not necessary to account for the phenomena we have addressed. While it would be possible to endogenize the *w* parameter, such that its value is determined entirely by properties of the private attitudes and social norms, we opted not to do so as we believe it has a natural interpretation as an independent construct. For example, we speculate that some cross-cultural differences may be captured by the *w* parameter, such as differing relative emphasis on social harmony as opposed to tolerance of individualism (Markus & Kitayama, 1991); exploration of such links is however beyond the scope of the present paper. In addition, as noted above, *w* may vary for different attitudes (e.g., in terms of how central they are to identity).

### Implications

Finally, we note some broader implications of SST. There is wide concern surrounding “information bubbles” and internet-facilitated personalization (Pariser, 2011; Sunstein, 2007), and it has been suggested that “new media” make extreme views more sustainable (Glaeser et al., 2005). Although we have noted important qualifications to some of the more general claims that have made in this area, SST offers one account of the psychological mechanisms that may underpin such processes when they do occur. Developments such as the greater availability and size of the internet, or increasing numbers of ever more specialized TV channels, make it easier for individuals to find expressions of opinion similar to their own (such as extreme political blogs), and according to SST this provides an environment in which they can express views closer to their own (thus gaining authenticity-related utility) without suffering social extremeness aversion. Thus SST can explain how the ever more universal availability of information, as with the growth of the internet, may lead not to a coming-together of opinions and attitudes across societies as was originally predicted, but instead, at least in Anglicised Western nations, sometimes to precisely the opposite. SST specifies a mechanism by which exposure to conflicting viewpoints and alternative social norms should act against polarization (cf. the Contact Hypothesis: Allport, 1954/1979).

Our approach also speaks to wider debates about the relationship between preferences, choices, and happiness. How should policy-makers identify policies that maximize the well-being, or utility, of a society? According to a dominant strand within economics, the “revealed preferences” tradition (Samuelson, 1938), people’s preferences can only be inferred from their choices and hence one approach is to choose policies that allow people to have what they want as revealed by their choices. However, there are many well-known problems with such an approach (Hausman, 2011; Hausman & McPherson, 2006), and hence various alternatives have been offered (Bernheim & Rangel, 2009; Sugden, 2004). SST offers a psychological process framework within which to interpret a distinction between “decision utility” (what agents maximize when they choose what attitude to express) and “true utility” (the extent to which authentic preferences are satisfied). Given this distinction, we cannot infer what maximizes an individual’s “true” or “underlying” utility from their choice of attitude to express.

In summary, SST offers an account of polarization and a number of related phenomena within an agent-based modeling framework. SST’s core assumption is that agents’ choices of attitudes to express represent a utility-maximizing compromise between the competing demands of preferences for authenticity on the one hand, and aversion to social extremeness on the other. It is this assumption, together with the idea that attitudes and descriptive social norms must be represented as distributions rather than single points, that underpins the key behaviors of the model.

## Supplementary Material

10.1037/rev0000342.supp

## Figures and Tables

**Table 1 tbl1:** List of Demonstrations With Associated Parameters

Demonstration	Effect	*w*	γ
1.1	Social norm effects	varies	20
1.2	Norm consensus effects	.5	20
1.3	Backfire effects	.3 and varies	20
2.1	Polarization	.5 and varies	20 and varies
2.2	Network well-being	.5	20
2.3	Social contagion	.8	varies
2.4	False consensus	.5	20
2.5	Pluralistic ignorance	.9	1
S1	Relative rank effects	n/a	15
S2	Changing private attitudes	.5 and varies	20
S3	Simulated media influence	varies	20

**Table 2 tbl2:** Network Behavior Under Different Parameter Values

Parameters	Disutility (*SD*)	False consensus (*SD*)	Polarization (*SD*)
γ = 5	896.59 (2.14)	0.99 (0)	1.54 (0.01)
γ = 10	81.3 (0.8)	0.99 (0)	1.96 (0.02)
γ = 20	0.77 (0.04)	1 (0)	2.24 (0.02)
γ = 50	0 (0)	1 (0)	2.39 (0.02)
*w* = .01	0.55 (0.01)	0.99 (0)	1.22 (0.00)
*w* = .30	0.76 (0.11)	0.99 (0)	1.94 (0.01)
*w* = .70	3.54 (0.76)	0.99 (0)	2.91 (0.04)
w = .99	1.01 (0.06)	0.93 (0)	12.78 (0.53)
α = 3; β = 3	13.7 (1.99)	0.98 (0)	1.78 (0.02)
α = 10; β = 10	0.77 (0.06)	1 (0)	2.24 (0.02)
α = 30; β = 30	0.57 (0)	0.99 (0)	2.22 (0.02)
α = 3; β = 10	2.97 (0.4)	0.98 (0)	2.08 (0.03)
α = 10; β = 3	2.95 (0.31)	0.98 (0)	2.08 (0.03)
*Note*. Parameter values are α = 10, β = 10, *w* = .5, and γ = 20 except as indicated in the left-most column. Measures of disutility and false consensus are taken after 50,000 cycles of the simulation; polarization is the ratio of the variances in mean attitudes after 50,000 cycles to the variance after the first cycle.			

## Footnotes

1 Descriptive social norms therefore contrast with injunctive social norms, which are concerned with beliefs about what people *ought* to do rather than what they *typically* do.

2 We use “utility” to refer to that quantity that, according to the model, people seek to maximize. We avoid terms such as “subjective well-being” or “happiness” both because well-being is a multidimensional construct and because we do not wish to assume conscious awareness of the maximized quantity. However, we do (in contrast to common usage of the term within economics) think of utility as a psychological, and potentially measurable, quantity.

3 The latter assumption—that Alison has no direct access to her own private attitudes—is not strictly required for the present demonstrations but resonates with the idea that we infer our own attitudes and preferences from our own behavior much as an observer does (Bem, 1967; Wilson, 2002).

4 This idea resonates with, and indeed our model can be seen as one possible implementation of, Kuran’s (1995) account of a tension between intrinsic and expressive utility.

5 The notion of attitude strength, as quantified here, is distinct from its importance (as stated by its holder) or its consequentiality for behavior (Howe & Krosnick, 2017).

6 We assume here, as with the previous equation, that expressed attitudes fall between the median of the social norm and the median of the private attitude. In other cases, the absolute values would need to be taken.

7 Recall that the network structure is not necessarily based on physical topography. Hence, agents can increase their utility by choosing to pay more attention to arguments or individuals’ views that are more consistent with their own—that is, the same mechanism can operate though attention to particular political blogs or media outlets rather than actual spatial relocation. In either case, agents’ behavior can be understood as motivated reasoning.

8 In the simulation, this was done using the MATLAB command betafit.

9 To reduce simulation time, only the attitudes of agents whose neighbors have changed are updated following a switch.

10 A color movie showing the emergence of polarization in the network over time is available at http://bit.ly/2qCR9tG.

11 There are of course many possible negative societal consequences of polarization as well; these lie outside the scope of the present paper.

12 Color movies showing social contagion, as well as its absence under different parameter values, in the network over time are available at http://bit.ly/2pkCcsj and http://bit.ly/2tg68rP.

13 With Weize Zhao.
