# Supplementary material for: Social Sampling and Expressed Attitudes: Authenticity Preference and Social Extremeness Aversion Lead to Social Norm Effects and Polarization
Source: Psychol Rev. 2022 Jan;129(1):18–48. doi: 10.1037/rev0000342 (PMC8908732; doi:10.1037/rev0000342)
Supplement: Supplementary file 1 [file rev0000342_sm.docx]

**Supplementary Online Material**

Social Sampling and Expressed Attitudes: Authenticity Preference and Social Extremeness Aversion Lead to Social Norm Effects and Polarization

Gordon D. A. Brown Stephan Lewandowsky Zhihong Huang

**Demonstration S1: Relative rank and disutility.** One key assumption of SST is that the disutility associated with expressing an attitude increases non-linearly with that atiitude’s extremity in a distribution (whether the distribution represents an agent’s private attitude or the social norm). In the case of the social norm, the intuition underlying the assumption is straightforward: One experiences the most disutility only if one is in the most extreme 5% to 10% of the social distribution (the exact numbers being determined by the value of the γ parameter; see main text Figure 4A). Provided an agent is in (say) the middle two quartiles of the social distribution, in contrast, they will experience little or no disutility due to social extremeness aversion and hence will not be motivated to change their behavior (i.e., the attitude that they express). We illustrate how this process may work — and offer some empirical evidence consistent with our assumption — using data from a resource dilemma experiment (Bilderbeck et al., 2014). Bilderbeck et al. examined how individuals changed their behavior from trial to trial as a function of their position in the social norm represented by other people’s behavior in the previous trial. Specifically, groups of four players chose, on each round of a repeated game, how many points (between 1 and 20) to harvest from a renewable resource. The initial level of the resource was 230; after each round it was reduced by the sum of the four players’ harvests and then replenished by a (noisy) number of points. It was therefore in players’ interests to restrain their harvests and conserve the resource, as no more harvesting would be possible once the resource was driven down.

The experiment examined how the amount a player chose to harvest was influenced by the amounts harvested by the other players on the previous round. Three of the four players were (unbeknownst to the fourth, who was the experimental participant) confederates, and their harvests were controlled by the computer to induce variation in the social norm.^[[1]](#footnote-1)^ The dependent variable of interest here was the change in harvest made by a participant on trial *n* as a function of their relative ranked position in the social norm on trial *n-1*. In intuitive terms, we might expect a participant who learned that their harvest was high in the social norm on trial *n-1* (e.g., they harvest 16; the other three players harvest 9, 10, and 12) to reduce their harvest on trial *n*, while a participant who learned that their harvest on trial *n* was low in the social distribution (e.g., they harvest 6; the other three players harvest 9, 10, and 12) would be likely to increase their harvest on trial *n*.

This pattern is exactly what was found, and a summary of the results is shown in Figure S1. (Results are binned and averaged across participants; individual data plots and further methodological details are available in Bilderbeck et al., 2014). The graph shows that participants tended to reduce their harvests when their harvest in the previous round of the game ranked high within the social norm, and tended to increase their harvests when the amount they took in the previous round ranked low within the social norm. It is evident that the change in behavior (here, change in amount harvested) increases non-linearly as social extremeness increases, just as assumed by SST. The solid line represents the exponential relation between utility loss and social extremeness assumed by SST (with γ = 15 and a scaling factor of 12).

The fit should be treated with caution, as the results come for an experiment involving a neuropharmacological manipulation and, as plotted, do not account for possible regression to the mean (see Bilderbeck et al., 2014 for analysis and discussion). Nonetheless, the results and model fit appear at least consistent with the idea that utility loss — and the resulting change in behavior — will be non-linear in rank as assumed by SST.

SST assumes that the same principle applies with regard to an agent’s own private attitude, in that an agent suffers relatively little loss of authenticity-related utility provided they are not expressing a view that is far out in the tail of the distribution that represents their own private attitude. We have kept the value of γ equal for private attitudes and social norms, as there is no need to do otherwise to capture the effects we examine, but this restriction could be relaxed if necessary.

We also note — but do not here explore — the possibility of individual differences in the γ parameter. Such differences would involve differences in *relative* sensitivity to different degrees of departure from authenticity or the social norm, rather than the overall sensitivity to social norms vs. authenticity, as the latter is represented by the *w* parameter.

**Demonstration S2: Allowing private attitudes to change.** The assumption that agents’ private attitudes are fixed at the start of the process of segregation and polarization, and remain unchanged throughout, is central to most of the results we report in the main text. In this demonstration we relax this assumption, allow private attitudes to align themselves gradually with expressed attitudes, and explore the effects of this alignment on polarization and on the distribution of private attitudes.

We do this by introducing a convergence mechanism that causes each agent’s private attitudes to move, on each time cycle of the simulation, slightly in the direction of the attitudes that the agent expressed at the end of the previous cycle.^[[2]](#footnote-2)^ Specifically, we start with the already-reported simulation of segregation and polarization (main text Demonstration 2.1). For our first convergence simulation, all parameter values remain the same as in Demonstration 2.1. One additional parameter (convergence rate) must be introduced. On each time cycle of the simulation we take the difference between each agent’s private attitude and the attitude they are expressing (as represented by the agent’s α and β parameters) and we add a proportion (specified by the convergence rate parameter) of this difference onto their private attitudes. This leads to private and expressed attitudes gradually coming into line with one another. We set the convergence rate parameter to .2.

Intuition suggests that the gradual convergence of private and expressed attitudes should lead to reducing, rather than increasing, polarization over time. This intuition is confirmed by the simulation, the key results of which are shown in Figure S2 (cf. main text Figure 13, which shows the behavior of the network when parameters are identical except for the inclusion of convergence). It is evident that polarization reduces throughout the simulation, whether measured by the attitudes expressed by given percentiles of the population (defined in terms of the extremity of the attitudes they express; left panel) or by the variance in expressed attitudes (right panel). As in main text Figure 13, lines represent the most extreme 5%, 20% and 40% of the population at either end of the attitude distribution. The tendencies for both false consensus and overall utility to increase over cycles of the simulation were similar to the behavior that was observed when convergence is not included (main text Figures 14 and 16) and hence are not shown here.

We also examined the final distribution of private attitudes in the learning model as a function of the social comparison parameter *w*. All other parameters remain as above, while *w* took values of .01, .30, .70, and .99. Figure S3 plots the (fitted, beta) distribution of private attitudes at the end of the 50,000 cycles of the simulation for each value of *w*. For reference, the distribution of mean attitudes at the start of the simulation (i.e., the beta(10,10) distribution from which those initial attitudes were drawn) is also shown on each panel. For all values of *w* the final distribution of attitudes is narrower than the initial distribution, reflecting a convergence of attitudes in the network overall when each individual agent tends to move towards the social norm in its neighborhood. Moreover, the final distribution of mean attitudes becomes narrower when the influence of the social norm is greater. We note, however, that behavior is parameter-dependent because the equilibrium state of the network depends on the relative time course of movement and attitude convergence. If the convergence parameter is very small, polarization will occur before there has been much convergence, whereas if the convergence parameter is large the agents’ attitudes will have moved towards each other before significant polarization has happened.

In summary, as expected, allowing private attitudes to move gradually into conformity with expressed attitudes leads to reducing, rather than increasing, polarization over time. This confirms that the assumption of fixed private/underlying attitudes is central to SST’s explanation of polarization. Indeed, given that social influence is generally assumed to act in the direction of increasing homogeneity of expressed options, the assumed existence of private attitudes that act as a countervailing force is essential in SST to explain why social comparison may actually increase polarization.

**Demonstration S3: Effects of media or opinion leaders.** The preceding demonstration (S2) explored the effects of allowing private/authentic attitudes to move gradually into conformity with expressed attitudes. In a final set of simulations we continue this exploration by investigating the consequences for private attitudes if every agent is exposed, on every time cycle, to a consistent set of opinions expressed by additional agents. This can be thought of as representing the exposure of every agent to the same set of expressed attitudes which could in turn be thought of as those promulgated by media sources or political leaders (Bail et al., 2018).

The simulations proceed exactly as in S2 above, except that throughout the entire simulation the four diagonal neighbors of every agent are replaced by agents who consistently express an attitude of either .9 (one condition of the simulation) or .1 (other condition). This simulates the consumption of highly partisan media from one or other side of the political spectrum. The results are shown in Figures S4 and S5. Figure S4 shows the expressed attitudes of the population over time, and reduction in attitude variance, when the “social media” agents consistently express an opinion of .1 (top two panels) or .9 (bottom two panels; main text Figure 12 shows the relevant comparisons). Figure S5 shows the final distributions of underlying attitudes in each case. It is clear that the addition of constant inputs to every agent’s opinion neighborhood has a strong effect; there is convergence of both private and expressed attitudes towards the attitudes displayed by the constantly-present agents.

**Demonstration S4: Alternative formulations of utility-extremeness function.** When calculating how well conformity preferences are met when a given attitude is expressed, extremeness is defined in terms of the area under the curve that represents the social norm. Specifically, “extremeness” is the area bounded by the expressed attitude and the median of the social norm (Figure 1). We denote this area as *H*. In the model as described so far, disutility increases as a convex function of *H* (Figure 4a). The same convex function describes how disutility increases as the expressed attitude differs from the median of the distribution that represents private attitudes. Intuition suggests that any monotonically increasing function will lead to cases where the utility-maximizing attitude to express will fall somewhere in between the median of the authentic attitude and the median of the social norm (always assuming that the function is the same for authenticity preference and social extremeness aversion), and hence that polarization would continue to be seen. To confirm these intuitions, we examined the effects on network polarization of changing the form of the function from convex to concave. Specifically, we replaced equation 3 with:

$Disutility={1-e}^{\left( -H \right)}$ (S1)

and re-ran the simulation with all parameters unchanged except for γ which was set to 5 (instead of 20) in order to prevent disutility from increasing too quickly as a function of *H* (if γ remains at 20, disutility is close to ceiling for any value of *H* greater than around .2).

The results we obtained were very similar to those obtained with the standard (convex) function, and as there were no qualitative differences we do not show the results graphically here. We also briefly examined the effect of reversing the relationship, such that expressing attitudes that were far from authentic attitudes and/or the social norm led to *increased* utility (a kind of “anticonformity”). As intuition suggests, this alternative utility/extremeness led to an unstructured network in which each agent attempts to become located as far away as possible from similar others while at the same time expressing a view as distant as possible from the median of their own authentic attitudes. Because we do not think this is a psychologically plausible model, we did not explore it further. An alternative model, in which authenticity preference was preserved but social extremeness was preferred rather than avoided, led as expected to an outcome where agents expressed attitudes that were close to their median attitudes but each occupied its own location and no segregation occurred.

(Figure S1)


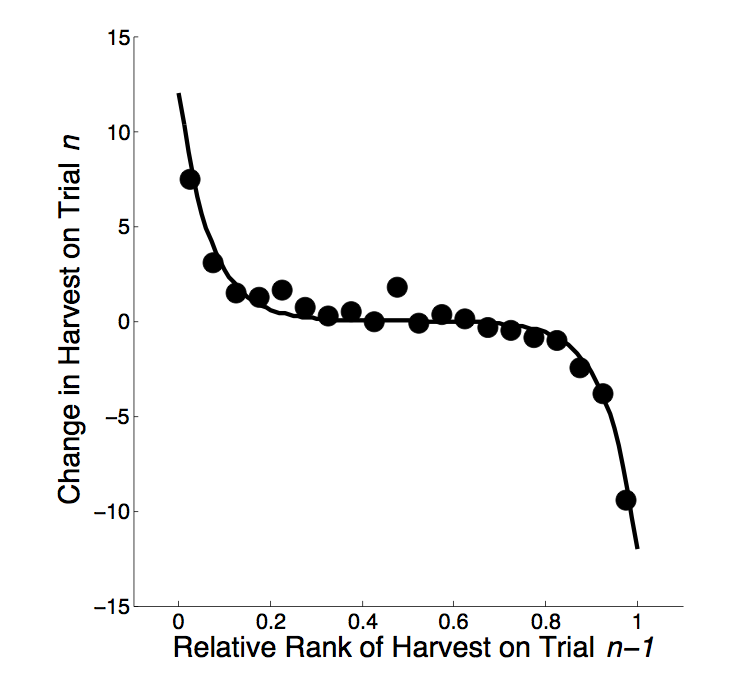


*Figure S1.* Non-linear behavior change as a function of ranked social extremeness on previous round.

(Figure S2)


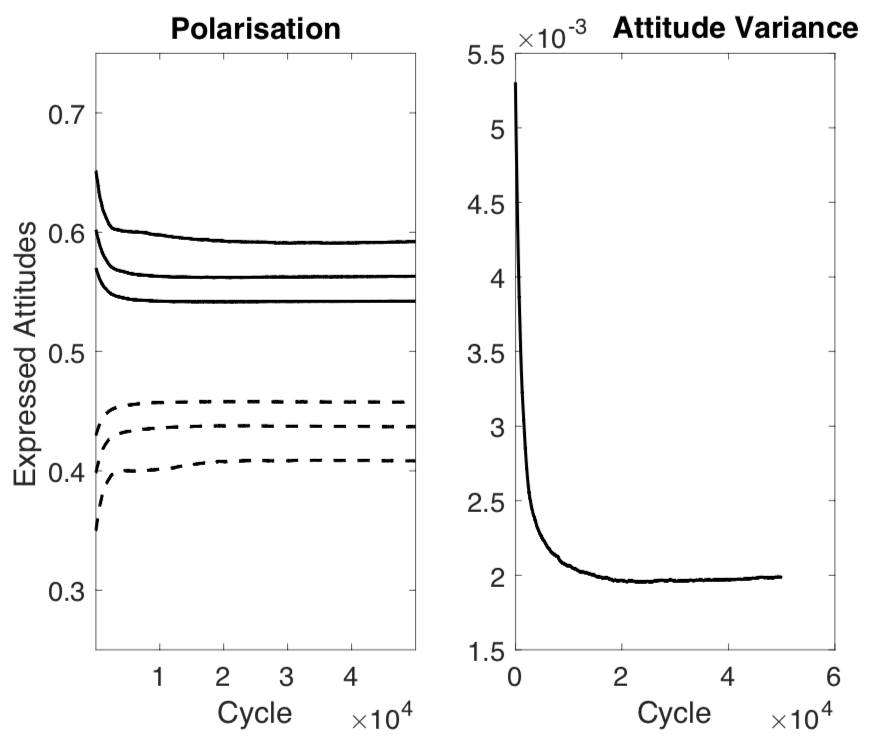


*Figure S2.* Left panel: evolution over time of the expressed attitudes of various percentiles of the population (with convergence between private and expressed attitudes). Right panel: Attitude variance increasing over time (with convergence between private and expressed attitudes).

(Figure S3)


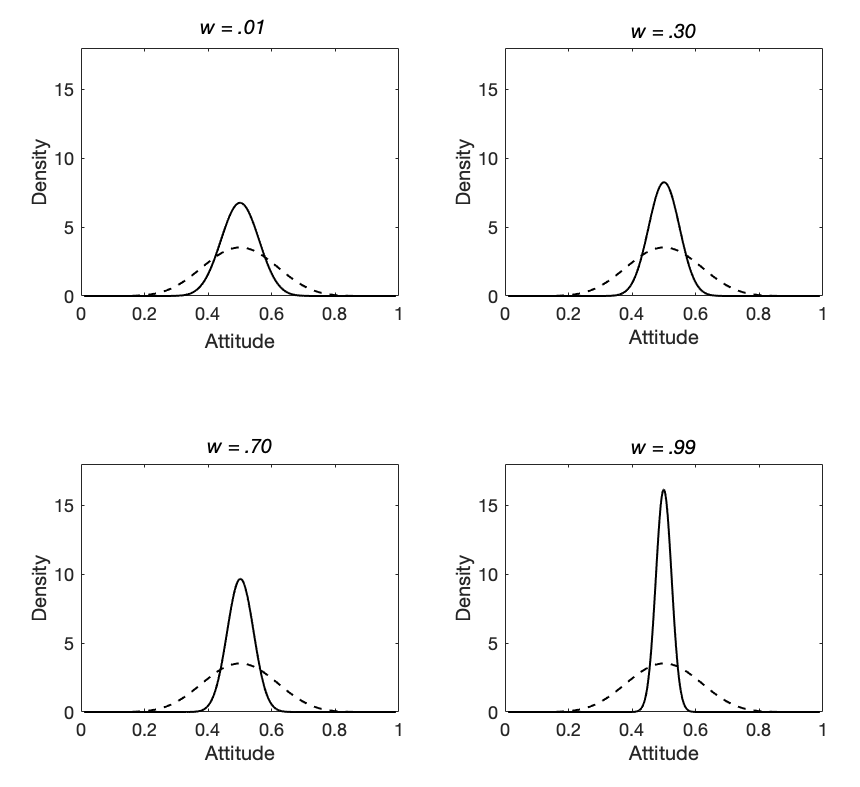


*Figure S3*. Final distributions (solid lines) of mean private attitudes after 50,000 cycles of learning for different values of *w* parameter. Initial distributions (dashed lines; same in each case) are shown for reference.

(Figure S4)


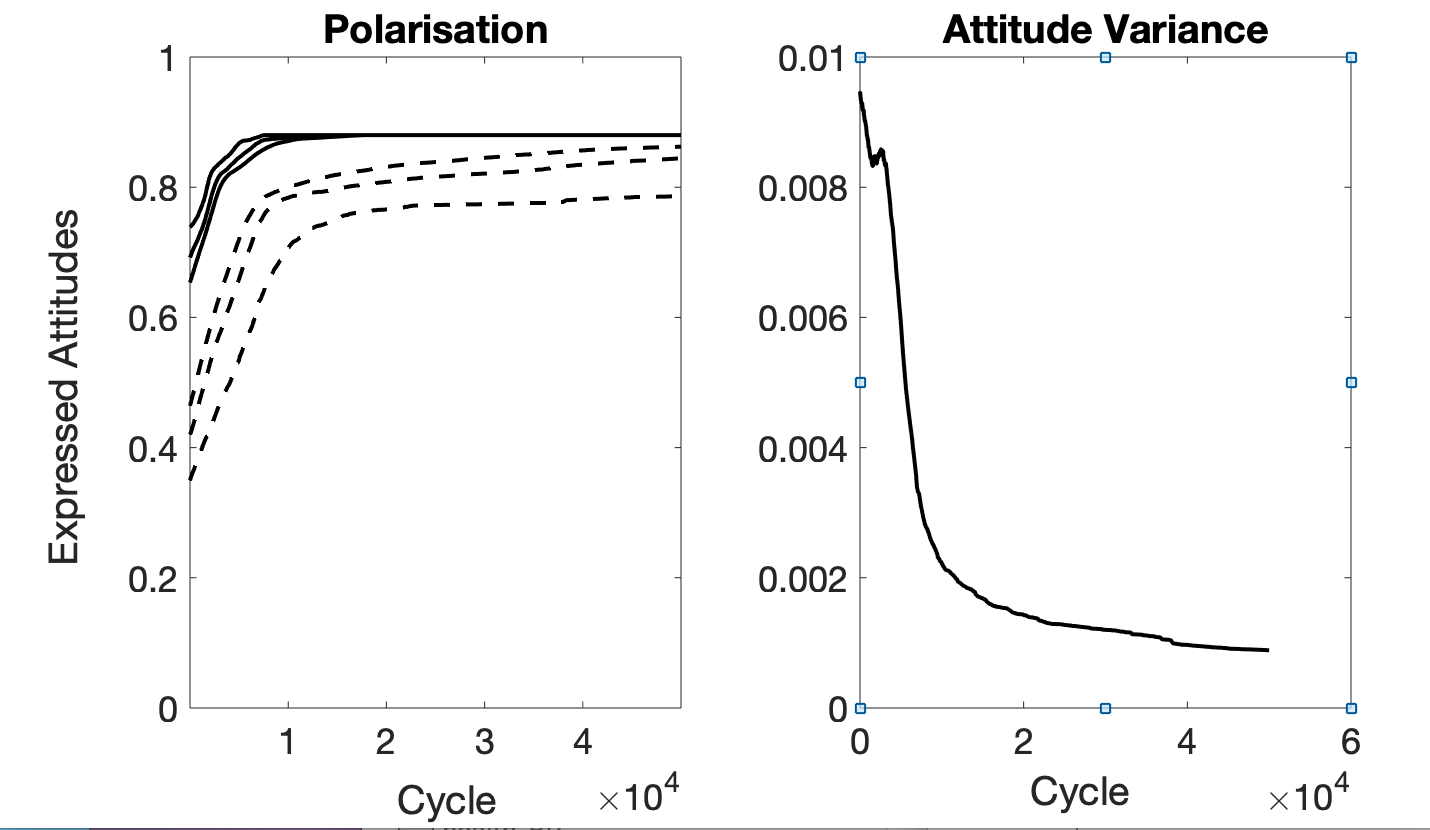


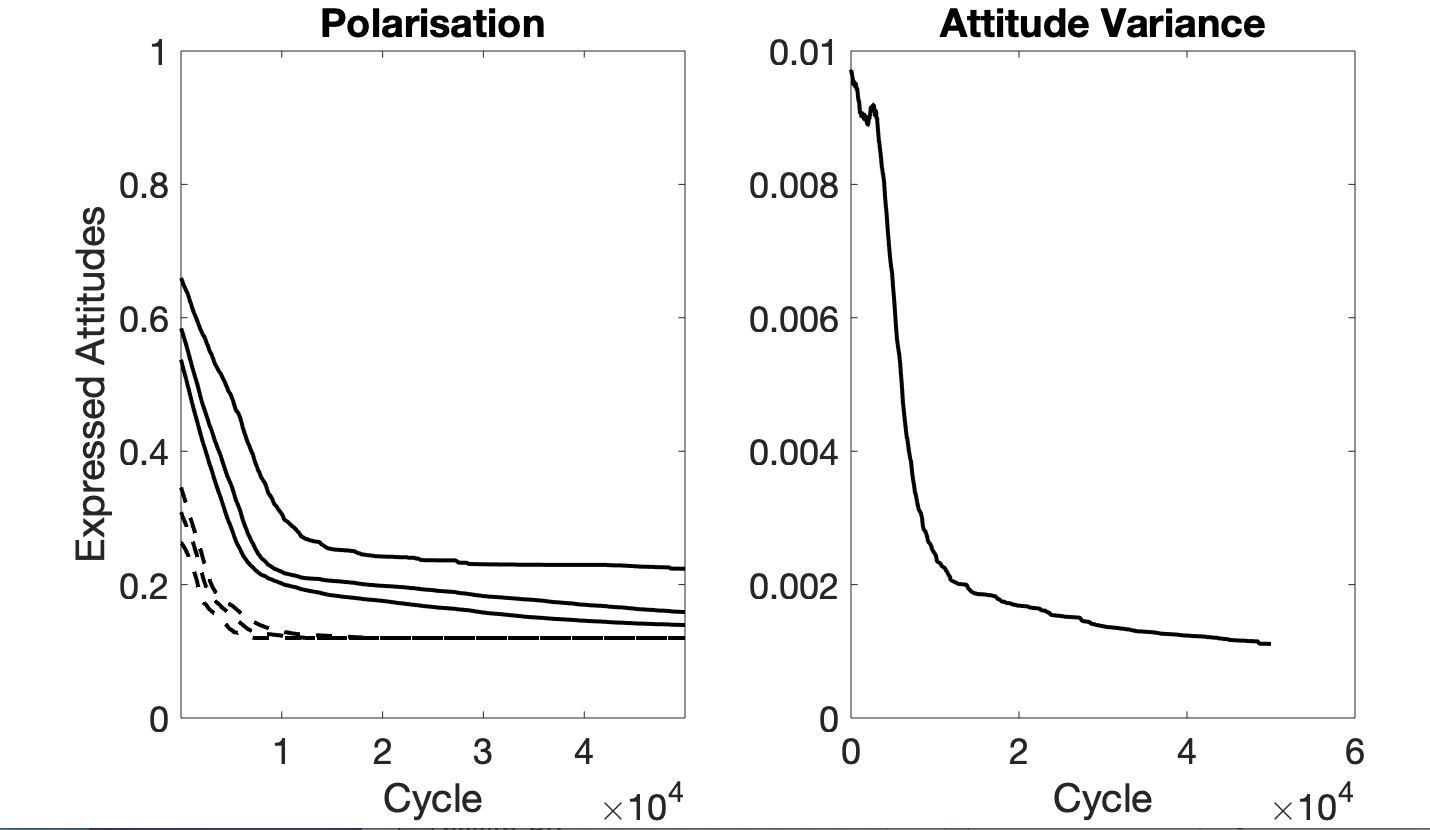


*Figure S4*. Left panels: evolution over time of the expressed attitudes of various percentiles of the population (with convergence between private and expressed attitudes). Right panels: Attitude variance increasing over time (with convergence between private and expressed attitudes). Top row: Simulated media consistently expresses .9; bottom row: Simulated media consistently expresses .1.

(Figure S5)


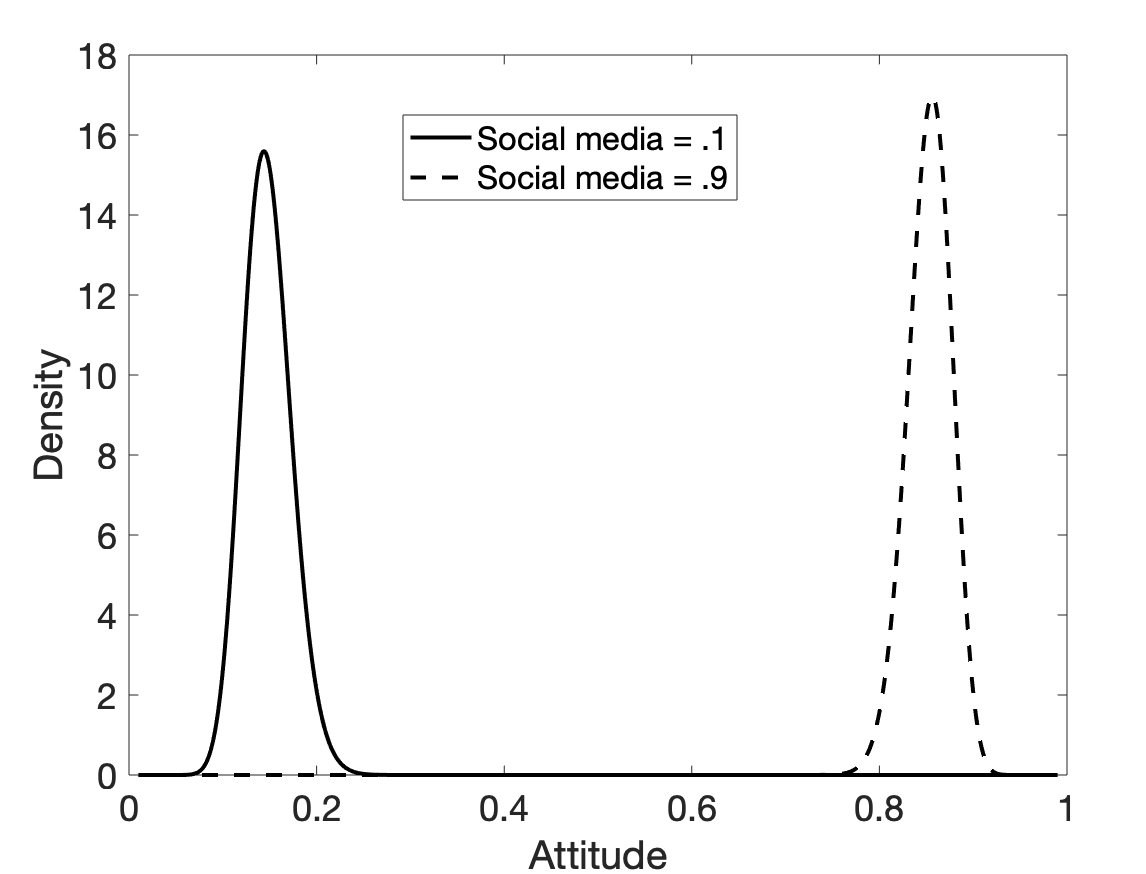


*Figure S5*. Final distributions of underlying attitudes after simulated exposure to consistent social media.

**References**

Bail, C. A., Argyle, L. P., Brown, T. W., Bumpus, J. P., Chen, H. H., Hunzaker, M. B. F., . . . Volfovsky, A. (2018). Exposure to opposing views on social media can increase political polarization. *Proceedings of the National Academy of Sciences of the United States of America, 115*(37), 9216-9221.

Bilderbeck, A. C., Brown, G. D. A., Read, J., Woolrich, M., Cowen, P. J., Behrens, T. E. J., & Rogers, R. D. (2014). Serotonin and social norms: Tryptophan depletion impairs social comparison and leads to resource depletion in a multiplayer harvesting game. *Psychological Science, 25*(7), 1303-1313.

1. All participants were tryptophan-depleted before the experiment to reduce serotonin activity; half of the participants drank an amino acid drink containing tryptophan before completing the experiment and only data from those participants are shown here. [↑](#footnote-ref-1)
2. As in attitude updating in the main text demonstrations, we only implement the learning process for agents in the neighborhood of other agents who have changed location. It is of course possible to allow convergence of all agents on every simulated cycle; this simply results in very rapid convergence and effectively places the polarization process (which involves only one exchange per cycle) on a much (and, we believe, implausibly) slower timescale than the learning/convergence. [↑](#footnote-ref-2)
